# Neural Markers Reveal a One-Segmented Head in Tardigrades (Water Bears)

**DOI:** 10.1371/journal.pone.0059090

**Published:** 2013-03-13

**Authors:** Georg Mayer, Susann Kauschke, Jan Rüdiger, Paul A. Stevenson

**Affiliations:** 1 Animal Evolution and Development, Institute of Biology, University of Leipzig, Leipzig, Germany; 2 Physiology of Animals and Behaviour, Institute of Biology, University of Leipzig, Leipzig, Germany; CNRS, France

## Abstract

**Background:**

While recent neuroanatomical and gene expression studies have clarified the alignment of cephalic segments in arthropods and onychophorans, the identity of head segments in tardigrades remains controversial. In particular, it is unclear whether the tardigrade head and its enclosed brain comprises one, or several segments, or a non-segmental structure. To clarify this, we applied a variety of histochemical and immunocytochemical markers to specimens of the tardigrade *Macrobiotus* cf. *harmsworthi* and the onychophoran *Euperipatoides rowelli*.

**Methodology/Principal Findings:**

Our immunolabelling against serotonin, FMRFamide and α-tubulin reveals that the tardigrade brain is a dorsal, bilaterally symmetric structure that resembles the brain of onychophorans and arthropods rather than a circumoesophageal ring typical of cycloneuralians (nematodes and allies). A suboesophageal ganglion is clearly lacking. Our data further reveal a hitherto unknown, unpaired stomatogastric ganglion in *Macrobiotus* cf. *harmsworthi*, which innervates the ectodermal oesophagus and the endodermal midgut and is associated with the second leg-bearing segment. In contrast, the oesophagus of the onychophoran *E. rowelli* possesses no immunoreactive neurons, whereas scattered bipolar, serotonin-like immunoreactive cell bodies are found in the midgut wall. Furthermore, our results show that the onychophoran pharynx is innervated by a medullary loop nerve accompanied by monopolar, serotonin-like immunoreactive cell bodies.

**Conclusions/Significance:**

A comparison of the nervous system innervating the foregut and midgut structures in tardigrades and onychophorans to that of arthropods indicates that the stomatogastric ganglion is a potential synapomorphy of Tardigrada and Arthropoda. Its association with the second leg-bearing segment in tardigrades suggests that the second trunk ganglion is a homologue of the arthropod tritocerebrum, whereas the first ganglion corresponds to the deutocerebrum. We therefore conclude that the tardigrade brain consists of a single segmental region corresponding to the arthropod protocerebrum and, accordingly, that the tardigrade head is a non-composite, one-segmented structure.

## Introduction

The composition of the arthropod head has been the subject of much controversy in the past and still remains one of the most contentious issues in the fields of comparative morphology and developmental biology today. Analysing the anterior body region in one of the closest relatives of arthropods, the Tardigrada, should provide useful insights for clarifying the ancestral head composition of Panarthropoda (Onychophora + Tardigrada + Arthropoda [1], [2]). The typical arthropod head is a composite structure consisting of several segments, which might have been added successively in the course of arthropod evolution [3]–[5]. The homology of the cephalic segments has been clarified recently in arthropods and onychophorans [6], [7], but the contradictory data from tardigrades [8]–[10] currently do not allow an adequate comparison with these two animal groups.

**Figure 1:**
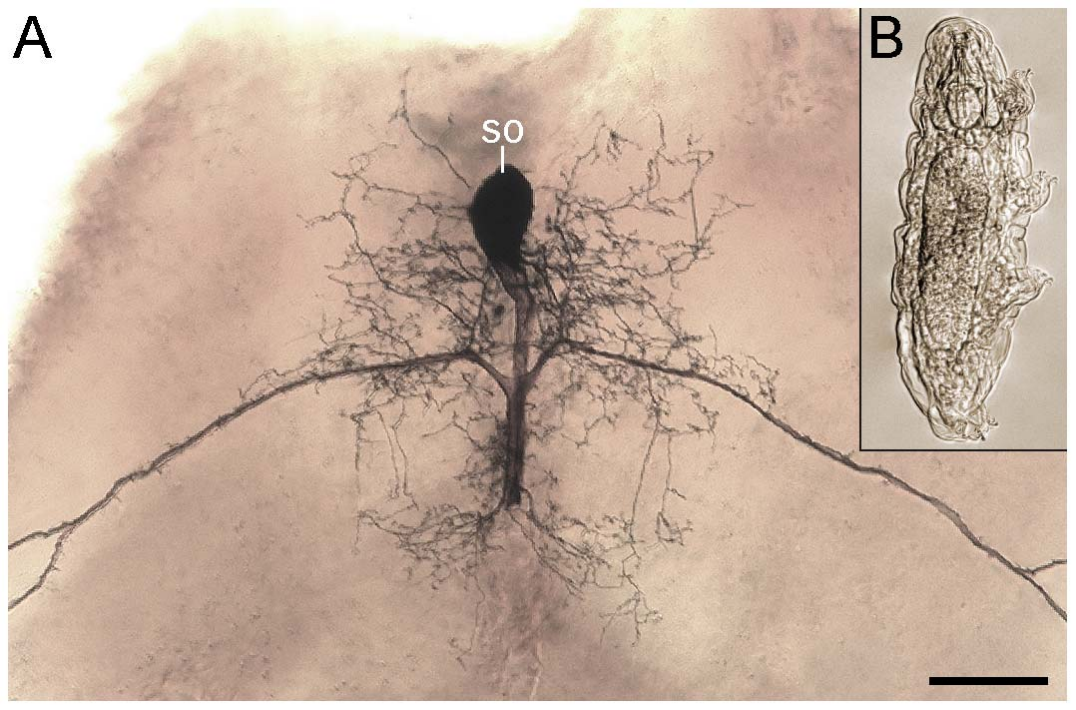
Relative size of a tardigrade compared to a single neuron of an insect. Light micrographs; both images are to scale. Note that the entire anterior end of the tardigrade, including the brain and consisting of hundreds of cells [79], would fit into a single neuronal cell body of the insect. (**A**) Cobalt-filled dorsal unpaired median neuron (DUM neuron) from the thoracic ganglion of the locust *Locusta migratoria*. (**B**) Specimen of *Macrobiotus* cf. *harmsworthi* in ventral view. Abbreviation: so, neuronal soma. Scale bar: 100 µm (for A and B).

The main problem in deciphering the organisation of the nervous system in tardigrades is due to their tendency to contract their bodies while being fixed, and their minute size, as for example the entire brain of the species studied herein would fit into the soma of a single insect neuron (Fig. 1A, B). Our understanding of the segmental composition of the tardigrade head depends on the unresolved issue of whether the tardigrade brain is a non-segmental structure or composed of one, two, or more segments [8]–[15]. The results of two recent immunocytochemical studies arrive at opposing conclusions, as they suggest that the tardigrade brain is either unsegmented and comparable to the ring-shaped brain of cycloneuralians [9], or tripartite (consisting of proto-, deuto- and tritocerebrum) and, therefore, similar to the brain of arthropods [10]. While both studies mainly focused on the distribution of commissures and neuropils in the tardigrade brain, this approach generally does not seem to be sufficient for resolving the number of segmental brain regions since a single segment can be composed of multiple neuropils [4]. Thus, it is essential to provide other lines of evidence in order to clarify the segmental composition of the tardigrade brain.

The position of the stomatogastric ganglia has been used successfully in the past to align the segmental regions of the brain and the head segments in arthropods [5], [16]. In most major arthropod groups, the stomatogastric ganglia, which innervate the ectodermal foregut and the endodermal midgut structures, are typically associated with the third body segment [5], [16]–[19]. Thus, clarifying the organisation and position of the stomatogastric nervous system in tardigrades might also help to align the anterior body segments between tardigrades and arthropods. Although a putative “stomatogastric” nervous system has been reported previously for tardigrades [15], the described innervation pattern does not correspond to the stomatogastric ganglia in arthropods, as the described neural elements exclusively innervate structures associated with the tardigrade mouth cone [8]. Additional nerves and neurites supplying the buccal apparatus, including the stylet and the anterior sensory structures, have been described in detail using confocal microscopy [9], [10], but the data on the innervation pattern of the midgut are still lacking in tardigrades.

To close this gap and to clarify the number of head segments in tardigrades, we applied a variety of cytochemical and immunolabelling markers in conjunction with confocal laser-scanning microscopy to visualise the nervous tissues of adult specimens of the limno-terrestrial tardigrade Macrobiotus cf. *harmsworthi*. For these experiments, we optimised our asphyxiation technique to obtain specimens with a fully extended body, which maximised the resolution of neuroanatomical structures in these minute animals (Figure 1B). For comparison, we analysed selected elements of the nervous system innervating the foregut and midgut structures in the onychophoran Euperipatoides rowelli. Our findings provide a clear framework for aligning the anterior body segments in tardigrades and arthropods.

## Materials and Methods

Specimens of Macrobiotus cf. harmsworthi Murray, 1907 (Eutardigrada, Macrobiotidae) were obtained from moss samples collected in the Volkspark Großdeuben near Leipzig (Saxony, Germany; N 51°14', E 12°23'). No specific permits were required for the collection of tardigrades since the location is not privately-owned or protected in any way and no endangered or protected species were involved. Specimens of Euperipatoides rowelli Reid, 1996 (Onychophora, Peripatopsidae) were obtained from rotten logs in the Tallaganda State Forest (New South Wales, Australia; S 35°26', E 149°33'). The necessary permits for the collection of onychophorans were obtained from the Forestry Commission of New South Wales, Australia (Special Purposes Permit for Research no. XX51212). Immunocytochemistry and vibratome sectioning were performed as described previously [20]–[24], except that most tardigrades were asphyxiated in hot water (75°C for 5–10 min) and their cuticle punctured with fine tungsten pins prior to fixation (in 4% paraformaldehyde in 0.1M phosphate-buffered saline [ = PBS], pH 7.4, for 6–12 hours). Incubations with antibodies were carried out either overnight or for several days. The following primary antibodies were used:

As general markers of neural structures, we used two different antibodies that both stain α-tubulin, a major component of axonal processes, either separately or in combination to increase the intensity of labelling. One antibody, anti-tyrosinated α-tubulin (Sigma-Aldrich, St. Louis, MO, USA; diluted 1:200), is a monoclonal antibody raised in mice against a synthetic peptide (T13) containing 11 C-terminal amino acids of α-tubulin from porcine brain plus an additional N terminal lysine and a C-terminal tyrosine at the C-terminus [25], [26]. This antibody has been used successfully to label neuron fibres in various metazoans, including cnidarians [27], acoelans [28], annelids [29] and chaetognaths [30]. The second antibody, anti-acetylated α-tubulin (Sigma-Aldrich; diluted 1:500), is also a mouse monoclonal antibody, but directed against acetylated α-tubulin from the outer arm of the sea urchin Strongylocentrotus purpuratus. This antibody recognises an epitope located on the α3 isoform of Chlamydomonas axonemal α-tubulin, within four residues of Lys^40^ when this amino acid is acetylated [31] and has been used to detect acetylated α-tubulins from many organisms, including onychophorans [4], [32], [33].

In addition, we used two antisera that recognise different neuromodulators that are common in invertebrates. Anti-FMRFamide (Incstar, Stillwater, MN, USA; currently ImmmunoStar, Hudson, WI, USA; diluted 1:1000) is a polyclonal antibody raised in rabbits against the neuropeptide FMRFamide coupled to bovine thyroglobulin. Its specificity has been established by the manufacturer and it has been used to label FMRFamide containing neurons in a wide variety of invertebrates, including cnidarians [34], acoelomorphs [35], rotifers [36], molluscs [37], annelids [38] and numerous insects (e.g., ref. [39]). We refer to the labelled structures in our specimens as “RFamide-like” immunoreactivity since the antibody labels a variety of peptides terminating with the sequence RFamide. Anti-serotonin (NT 102 Eugene Tech Inc., NJ, USA; currently Protos Biotech, NJ, USA; diluted 1:1000) is a polyclonal antiserum raised in rabbits against the biogenic amine serotonin, coupled to Limulus haemocyanin. Its specificity has been established and it has been shown to recognise serotonergic neurons for example in molluscs [40], insects [41] and rats [42]. Since we cannot fully exclude that the antiserum may bind to serotonin-related substances, in addition to serotonin, we refer to the observed labelling as “serotonin-like” immunoreactivity.

All antibodies were diluted in 1% normal goat serum in 0.1 M PBS, pH 7.4, containing 1% Triton-X. Bound antisera were detected using Alexa 488- or 568-tagged secondary antibodies (Invitrogen, Carlsbad, CA, USA) diluted 1:500. After several rinses in PBS, some specimens and sections were incubated for one hour in a solution containing phalloidin-rhodamine (Invitrogen) to stain f-actin as described previously [32]. After additional rinses in PBS, the DNA-selective fluorescent dyes Hoechst (Bisbenzimide, H33258; Sigma-Aldrich; 1 μg/ml in PBS) or SYBR® Green (Invitrogen) were applied according to the manufacturers’ protocols. Specimens were then rinsed in PBS and mounted between two cover slips in Vectashield mounting medium (Vector Laboratories Inc., Burlingame, CA, USA) or dehydrated in an ethanol series and mounted in methyl salicylate.

All preparations were analysed with the confocal laser-scanning microscopes Zeiss LSM 510 META (Carl Zeiss MicroImaging GmbH, Jena, Germany) and Leica TCS STED (Leica Microsystems, Wetzlar, Germany). Confocal image stacks were processed with Zeiss LSM IMAGE BROWSER v4.0.0.241 (Carl Zeiss MicroImaging GmbH), Leica AS AF v2.3.5 (Leica Microsystems), and IMARIS 7.2.1 (Bitplane, Zurich, Switzerland). Scanning electron microscopy was carried out as described previously [24]. Final panels and diagrams were produced using Adobe (San Jose, CA, USA) Photoshop CS4 and Illustrator CS4.

## Results

### Basic architecture of the central nervous system in the tardigrade *Macrobiotus* cf. *harmsworthi*

**Figure 2:**
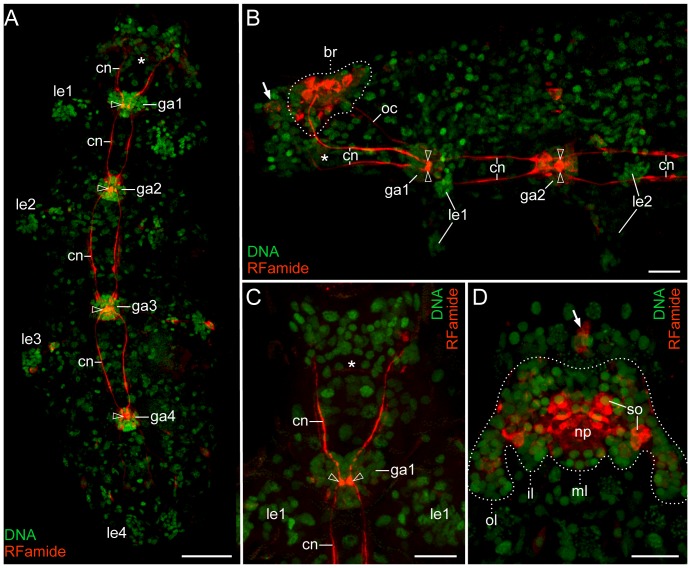
Organisation of the nervous system in the tardigrade Macrobiotus cf. *harmsworthi*. See also Figures S1, S2 for the colour-blind. RFamide-like immunoreactivity (red) and DNA labelling (green). Maximum projections of confocal z-series. Anterior is up in all images except for B in which anterior is left. Note that no “suboesophageal” or “subpharyngeal ganglion” is evident in its presumptive position (asterisks in A–C). Arrowheads in A–C point to varicose swellings in the core of each trunk ganglion. Arrows in B and D indicate two anterior, extra-cerebral RFamide-like immunoreactive cell bodies. (**A**) Specimen in ventral view showing four trunk ganglia linked by somata-free connectives. (**B**) Specimen in lateral view revealing the dorsal position of the brain (dotted line). (**C**) Anterior end in ventral view. (**D**) Anterior end in dorsal view with details of central brain neuropil. Dotted line indicates the shape of the brain with its lobes. Abbreviations: br, brain; cn, connectives; ga1–ga4, trunk ganglia 1 to 4; il, inner brain lobe; le1–le4, walking legs 1 to 4; np, central brain neuropil; oc, outer connective; ol, outer brain lobe; ml, median brain lobe; so, neuronal somata. Scale bars: 25 µm (A), and 10 µm (B–D).

**Figure 3:**
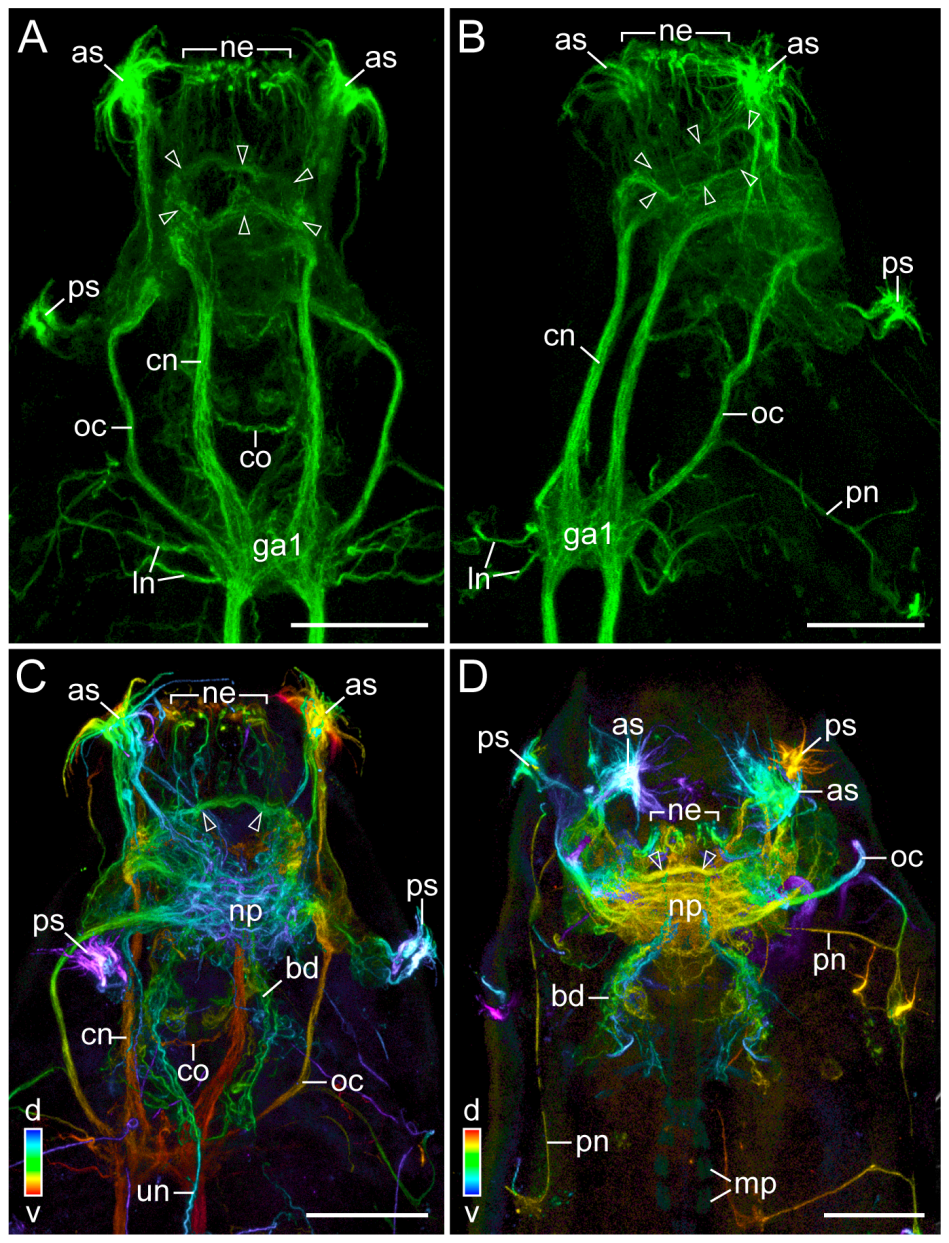
Neural structures in the anterior end of the tardigrade Macrobiotus cf. *harmsworthi*. Combined anti-tyrosinated and anti-acetylated α-tubulin immunolabelling. Anterior is up. Arrowheads point to the nerve ring, which gives rise to numerous anterior neurites innervating the peribuccal/mouth lamellae. (**A, B**) Specimens in ventral and ventrolateral view, respectively. Maximum projections of confocal z-series. (**C, D**) Depth-coded projections of confocal z-series of anterior ends of an extended (in C) and a contracted specimen (in D). Note the complex fibre network within the central brain neuropil, which appears as a unitary structure with the nerve ring in the contracted specimen. Abbreviations: as, anterolateral sensory fields; bd, neurite bundles innervating the stylet musculature; cn, connective; co, commissure of the stomodeal complex (cf. Figure 5C); ga1, first trunk ganglion; ln, leg nerves; mp, macroplacoids; ne, neurites supplying the peribuccal/mouth lamellae; np, central brain neuropil; oc, outer connective; pn, peripheral nerve; ps, posterolateral sensory fields; un, unpaired posterior nerve/neurite. Scale bars: 20 µm (A–D).

Confocal images of entire specimens of Macrobiotus cf. *harmsworthi* immunolabelled with a selection of antisera in combination with nuclear staining revealed a 200–250 µm long chain of four ventral trunk ganglia and a dorsally situated anterior brain (Figures 2A–D, 3A–D and Figures S1, S2). The brain and the four trunk ganglia are linked by paired, somata-free connectives. An additional pair of outer connectives joins the first trunk ganglion with the brain (Figure 3A–D). While the outer connectives are labelled with antibodies directed against α-tubulin, they show only weak RFamide-like immunoreactivity (Figures 2B, 3A–D and Figures S1, S2).

The four trunk ganglia are clearly discernible as aggregations of tiny nuclei (diameter ∼2 µm) of presumptive neurons that have a composite total diameter of 15–25 µm, positioned just anterior to each of the four pairs of legs, which can also be discerned by aggregations of nuclei (Figure 2A–C and Figure S1). Anti-tubulin immunolabelling reveals numerous contralateral projections and commissure-like structures within each ganglion and an additional extra-ganglionic commissure in front of the second, third and fourth trunk ganglia (Figure S3). We also observed vivid RFamide-like immunoreactive staining of two varicose swellings that lie in close apposition to each other in the core of each trunk ganglion (arrowheads in Figure 2A–C and Figures S1, S2). The shape of these structures differs between the ganglia of the same individual but is similar in the same ganglion of different specimens. Since they lack a nucleus they are clearly not somata. Their small size (maximum diameter 2.5 µm) is in the range of that of synaptic terminals. Since we observed no other clearly RFamide-like labelled neuropil structures in the trunk ganglia, these varicosities could possibly represent individual synaptic terminals in close association.

### Organisation of the brain and associated neural structures in the tardigrade *Macrobiotus* cf. *harmsworthi*

**Figure 4:**
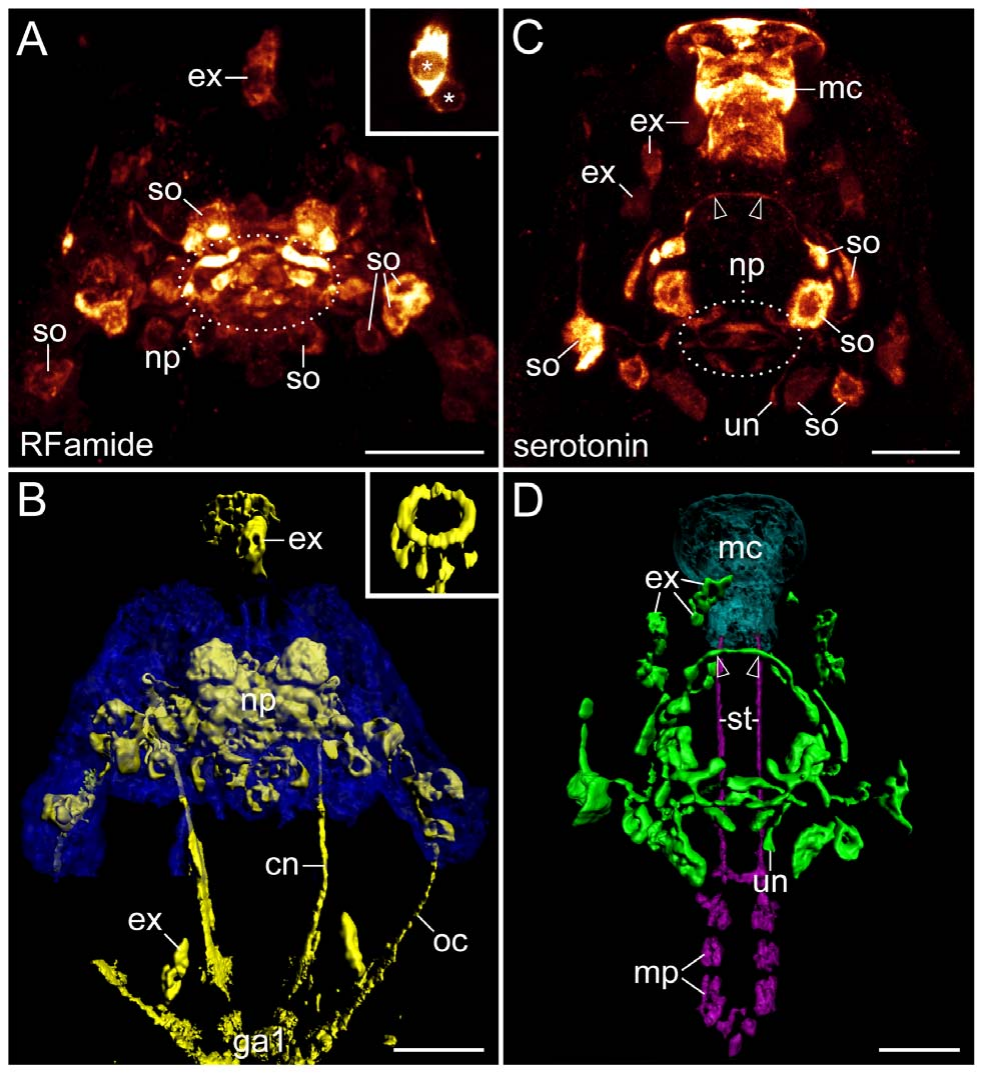
Details of the brain in the tardigrade Macrobiotus cf. *harmsworthi*. Specimens in dorsal view; anterior is up. Note the bilaterally symmetric arrangement of individually identifiable neuronal somata. (**A**) RFamide-like immunoreactivity; maximum projection of confocal z-series. Inset shows details of two extra-cerebral RFamide-like immunoreactive somata situated in front of the brain (asterisks). (**B**) Volume rendering of the same dataset as in A. The brain contours are illustrated in blue (autofluorescence), RFamide-like immunoreactive structures in yellow. Inset shows details of a ring-like structure surrounding the mouth. (**C**) Serotonin-like immunoreactivity; maximum projection of confocal z-series. Note the strong signal in the mouth cone. (**D**) Volume rendering of the same dataset as in C. Serotonin-like immunoreactive neural structures are illustrated in green, the mouth cone in light-blue, autofluorescent calcified stylet structures in magenta. Arrowheads in C and D point to two bilateral neurites, which join the dorsal portion of the ring nerve (see also Movie S2). Abbreviations: cn, connective; ex, cell bodies of extra-cerebral neurons; ga1, first trunk ganglion; mc, mouth cone; mp, macroplacoids; np, central brain neuropil; oc, outer connective; so, neuronal somata; st, stylet; un, unpaired posterior neurite/nerve. Scale bars: 10 µm (A–D).

DNA labelling and analyses of confocal z-series reveal that the brain of Macrobiotus cf. *harmsworthi* contains ∼200 nuclei that are arranged in a bilaterally symmetric pattern (Figure 2D and Figure S1). Since some of these nuclei are likely to be from non-neuronal cell types, such as glia cells, the tardigrade brain might contain fewer than 200 neurons. In shape, the brain appears lobate in dorsal view, as it consists of one pair of outer lobes, one pair of inner lobes and an unpaired median lobe. There is no obvious grouping of somata into clusters within the lobes. Anti-RFamide and anti-serotonin immunolabelling reveals somata of individually identifiable neurons of different size, which are arranged in a bilaterally symmetric pattern (Figure 4A–D). In all we could discern some 14 serotonin-like and ∼26 RFamide-like immunoreactive neurons within the brain (Movies S1 and S2).

In addition, two extra-cerebral RFamide-like immunoreactive cell bodies are found dorsally behind the brain (Figure 4B and Movie S1). Each of these two somata sends out a neurite, which enters the brain between the inner and median brain lobes (Movie S1). Additional two RFamide-like and ten serotonin-like immunoreactive somata are located anterior to the brain (Figure 4A–D). The two RFamide-like immunoreactive cell bodies cluster together in the dorsomedian head region (inset in Figure 4A). The anterior cell body gives rise to two lateroposterior neurites and to an unpaired anterior neurite, which forms a ring-like structure around the mouth (inset in Figure 4B), whereas the weakly labelled posterior cell body sends out a single posterior neurite towards the brain (Movie S1). The ten extra-cerebral, serotonin-like immunoreactive cell bodies are not associated with the brain but are spatially distributed around the mouth cone. One lies dorsomedially, one ventromedially, two are closely associated with the lateral portions of the buccal tube and three bilateral pairs lie further posteriorly (Figure 4C, D, Figure S4 and Movie S2). The ten extra-cerebral, serotonin-like immunoreactive somata send single neurites to the buccal tube. Notably, the distal portion of the buccal tube is strongly immunoreactive against serotonin and this peculiar labelling was observed in all specimens labelled against serotonin (n = 12).

The central brain neuropil, as revealed by RFamide-like, serotonin-like and α-tubulin immunoreactivity, appears as a complex unitary structure comprising numerous conglomerates of immunoreactive fibres and commissures, which may represent specialised neuropils (Figures 2D, 3C, D, 4A–D, Figures S1, S2 and Movies S1–S3). The lateral portions of the central neuropil receive fibres from the anterior-most and outer connectives, which link the brain to the first trunk ganglion (Figure 3C, D). In addition, single fibres and bundles of neurites extend from the paired anterolateral and posterolateral sensory fields to the central brain neuropil (Figure 3A–D).

**Figure 5:**
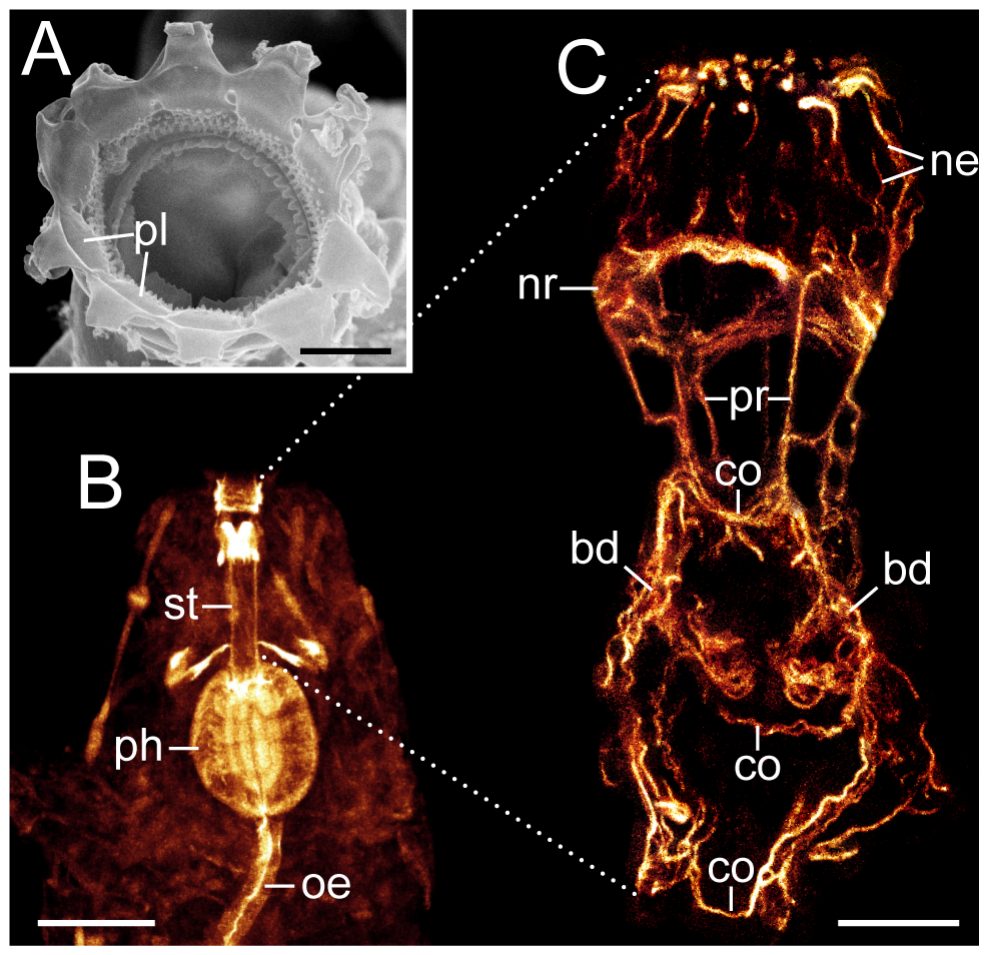
Details of the stomodeal innervation in the tardigrade Macrobiotus cf. *harmsworthi*. (**A**) Scanning electron micrograph showing the peribuccal lamellae surrounding the mouth opening. (**B**) Anterior end of a specimen labelled with phalloidin-rhodamine to reveal the stylet and pharyngeal musculature. Projection of a confocal z-series; anterior is up. (**C**) Stomodeal complex. Combined anti-tyrosinated and anti-acetylated α-tubulin immunolabelling. Projection of confocal z-series; anterior is up. For the sake of clarity, only elements of the stomodeal complex were selected from each optical section for the projection. Dotted lines demarcate the corresponding body region in the specimen labelled with phalloidin-rhodamine. Abbreviations: bd, neurite bundles innervating the stylet musculature; co, commissures of the stomodeal complex; ne, neurites supplying the peribuccal/mouth lamellae; nr, nerve ring; oe, oesophagus; ph, pharynx; pl, peribuccal/mouth lamellae; pr, projections linking the nerve ring and the neurite bundles innervating the stylet musculature; st, stylet. Scale bars: 3 µm (A), 25 µm (B), and 10 µm (C).

**Figure 6:**
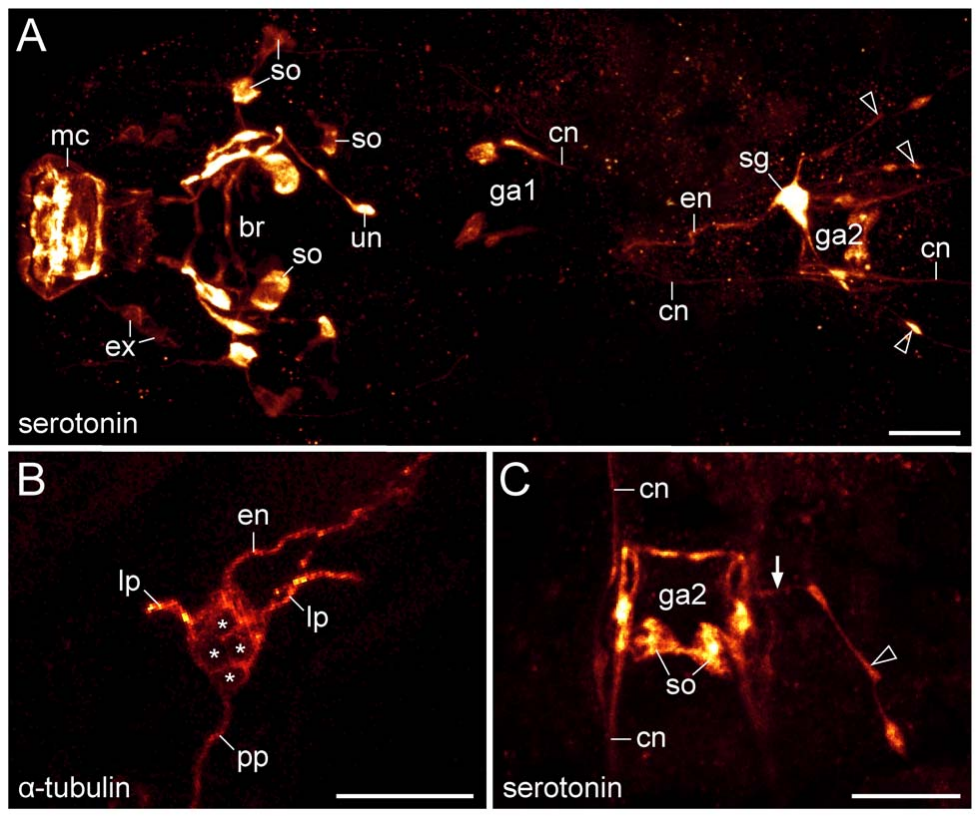
Characteristics of the stomatogastric ganglion supplying the oesophagus and midgut in the tardigrade Macrobiotus cf. *harmsworthi*. Projections of confocal z-series. (A) Anterior half of a specimen in dorsal view. Anti-serotonin immunolabelling. Maximum projection of a confocal z-series. Note serotonin-like immunoreactivity in the stomatogastric ganglion. Arrowheads point to three posterior neurites. (B) Detail of the stomatogastric ganglion. Combined anti-tyrosinated and anti-acetylated α-tubulin immunolabelling. Neuronal cell bodies are marked by asterisks. (C) Detail of a lateral posterior neurite (arrowhead), which has an input into the second trunk ganglion (arrow). Anti-serotonin immunolabelling. Projection of a confocal z-series. Abbreviations: br, position of the brain; cn, connective; en, oesophageal neurite; ex, somata of extra-cerebral neurons innervating the mouth cone; ga1–ga2, position of the first and second trunk ganglia; lp, lateral neurite; mc, mouth cone; pp, posterior neurite; sg, stomatogastric ganglion; so, neuronal somata; un, unpaired posterior neurite/nerve. Scale bars: 10 µm (A–C).

In front of the central neuropil, there is a prominent ring nerve surrounding the buccal tube (Figure 3A–C and Figure S5). This nerve receives fibres from the brain as well as from the two connectives linking the brain to the first trunk ganglion (Figures 3A–C and Movie S3). The ring nerve gives rise to numerous neurites projecting anteriorly to the peribuccal lamellae surrounding the mouth opening (Figure 3A–C, 5A–C, Figure S5 and Movie S3). Moreover, it receives fibres from bundles of neurites innervating the stylet apparatus (Figure 5B, C). These bundles arise ventrally from the central brain neuropil and project posteriorly to innervate the musculature of the stylet apparatus (Figures 3C, 5B, C, Figure S6 and Movie S3). The bundles are linked by three ventral commissures, whereas anteriorly they send off several longitudinal projections that join the ring nerve (Figure 5C). Anti-α-tubulin and anti-serotonin immunolabelling reveals an unpaired posterior nerve, which originates from the brain and passes through the stylet bundle further into the wall of the pharynx (Figures 3C, 6A, D and Figure S6). Anti-α-tubulin immunolabelling shows additional fibres associated with this nerve, which give rise to a loop-like structure within the pharyngeal wall (Figure S6).

Although our data show that the ring nerve and the central brain neuropil are clearly distinguishable in extended specimens (Figure 3C), they appear as a fused, unitary structure in contracted specimens (Figure 3D). Similarly, the anterior neurites innervating the peribuccal lamellae surrounding the mouth are clearly visible in extended specimens but obscured after contraction (Figure 3C, D and Figure S7). The extent of distortion of neural structures situated in the head is particularly evident in the position of the anterolateral and posterolateral sensory fields, which are widely separated in fully extended specimens, but in close proximity in contracted animals.

### No evidence of a “suboesophageal”/“subpharyngeal ganglion” in the tardigrade *Macrobiotus* cf. *harmsworthi*

Notably, the central neuropil and all cell bodies of RFamide-like and serotonin-like immunoreactive neurons of the brain in Macrobiotus cf. *harmsworthi* occur dorsally and dorsolaterally to the buccal tube, while ventrally no neurons are found (Figures 2A–D, 4A–D, Figures S1, S2 and Movies S1, S2). The fibres of the anterior connectives from the first trunk ganglion circumvent the buccal tube via the ring nerve and connect with the dorsal brain, without condensing to form any additional neuropil structure or traversing any further conglomeration of nuclei (Figures 2B, C, 4A, B, Figures S1, S2 and Movie S1). Corresponding findings were obtained for 21 critically inspected preparations immunolabelled with an anti-FMRFamide antibody. The same holds true for other markers used, including α-tubulin and serotonin-like immunoreactivity (Figures 3A–C, 4C, D and Movies S2, S3). Thus, we found no indication whatsoever for the existence of a structure, which could in any way be regarded as representing a “suboesophageal” or “subpharyngeal ganglion”.

### Innervation of the oesophagus and midgut in the tardigrade *Macrobiotus* cf. *harmsworthi*

Anti-α-tubulin and anti-serotonin immunocytochemistry revealed hitherto unknown elements of the stomatogastric nervous system in Macrobiotus cf. *harmsworthi* (Figure 6A–C). Apart from neural structures innervating the mouth cone, the stylet apparatus and the pharynx, we detected a prominent ganglion ( = stomatogastric ganglion) located within the second leg-bearing segment, at the junction between the ectodermal oesophagus and the endodermal midgut (Figure 6A, B). This ganglion comprises at least four cells and sends off four neurites: three posterior neurites, which project along the wall of the midgut, and an anterior neurite, which runs along the oesophagus and ends blindly behind the pharynx (Figures 6A, B and Figure S8). Serotonin-like immunoreactivity reveals a connection of the lateral posterior neurites to the second trunk ganglion (Figure 6C).

### Innervation of the pharynx, oesophagus and midgut in the onychophoran *Euperipatoides rowelli*

**Figure 7:**
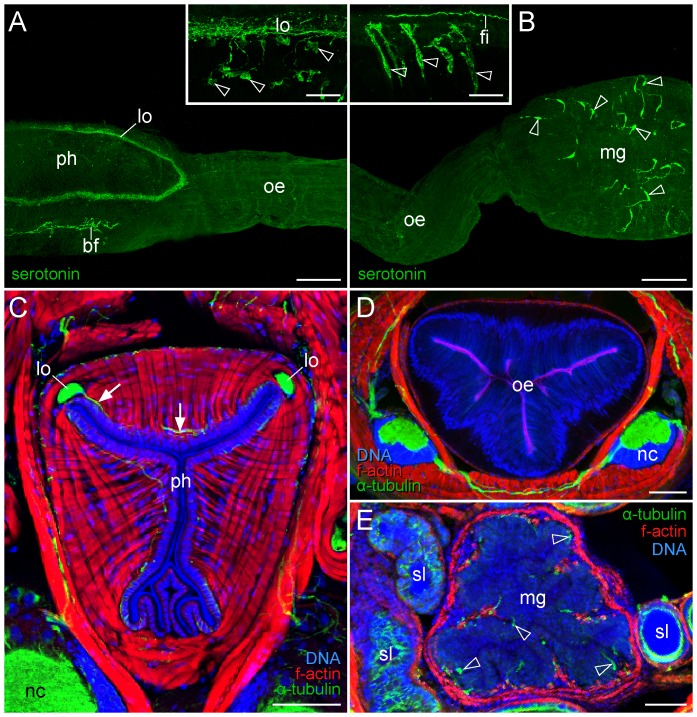
Innervation pattern of pharynx, oesophagus and midgut in the onychophoran Euperipatoides rowelli. See also Figure S9 for the colour-blind. Confocal micrographs. (**A, B**) Anti-serotonin immunolabelling of a dissected digestive tract. Anterior is left. (**C–E**) Triple-labelled vibratome cross-sections using an anti-acetylated α-tubulin antibody (green), phalloidin-rhodamine (red), and the DNA-selective dye Hoechst (blue). Dorsal is up. (**A**) Whole-mount preparation showing the pharyngeal loop nerve (lo). Inset, which is a projection of only a few focal planes, illustrates numerous serotonin-like immunoreactive somata associated with this nerve (arrowheads). (**B**) Detail of transition region between oesophagus and midgut in the same whole-mount preparation as in A. Note the scattered arrangement of neuronal cell bodies within the gut wall (arrowheads). Inset, which is a projection of only a few focal planes, shows a nerve fibre and details of serotonin-like immunoreactive somata of bipolar neurons within the gut wall (arrowheads). (**A–E**) Vibratome cross-sections of the pharynx (ph), oesophagus (oe) and midgut (mg) showing their innervation pattern. Arrows in C point to fibres supplying the pharyngeal musculature. Arrowheads in E indicate single neurons in the wall of the gut. Note the lack of nerves and neuronal cell bodies in the oesophagus wall in D. Abbreviations: bf, bundle of fibres in the pharyngeal wall; fi, serotonin-like immunoreactive fibre on the outer surface of the gut wall; lo, pharyngeal loop nerve; mg, midgut; nc, nerve cord; oe, oesophagus; ph, pharynx; sl, slime glands. Scale bars: 100 µm (A–E), and 25 µm (insets in A and B).

**Figure 8:**
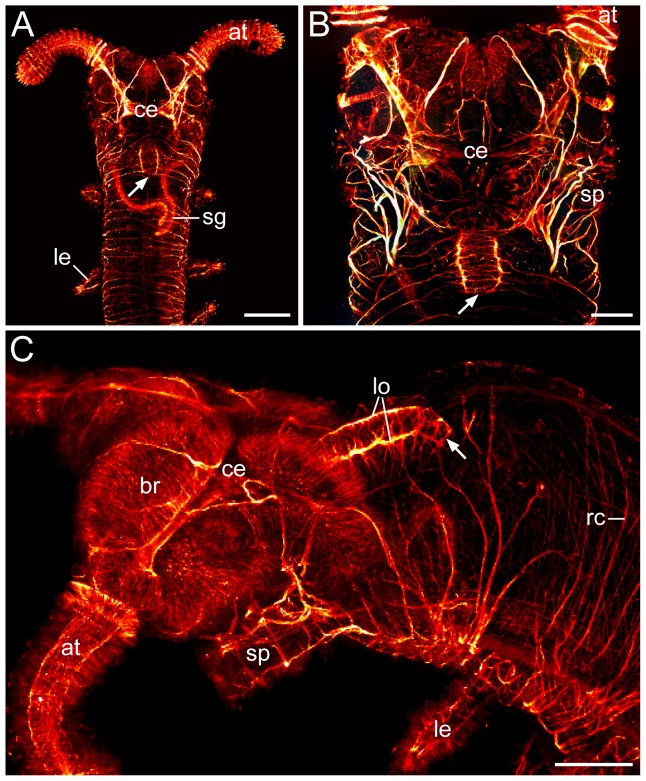
Innervation of the pharynx in embryos of the onychophoran Euperipatoides rowelli. (**A–C**) Whole-mount preparations of almost fully developed embryos (stage 7) in dorsal (in A and B) and lateral views (in C). Maximum projections of confocal z-series; anti-acetylated α-tubulin immunolabelling. Note that there is no nerve tract connection between the pharynx and the midgut (arrows). Abbreviations: at, antenna; br, brain; ce, central brain neuropil; le, leg; lo, pharyngeal loop nerve; rc, ring commissure; sg, slime gland; sp, slime papilla. Scale bars: 300 µm (A), and 100 µm (B, C).

In the onychophoran Euperipatoides rowelli, two distinct neural complexes innervate the foregut and midgut structures. Firstly, a paired nerve originating from the brain runs posteriorly in each dorsolateral corner of the pharynx and gives off fibres supplying the pharyngeal musculature (Figure 7A, C and Figure S9). This paired nerve unites posteriorly, thus forming a loop near the border of the pharynx and the oesophagus (Figures 7A, 8A–C and Figure S9). Notably, our data show that this pharyngeal nerve has a medullary organisation since it is accompanied by somata of monopolar, serotonin-like immunoreactive neurons, which send fibres into the nerve (inset in Figure 7A and Figure S9). Apart from this nerve, an additional bundle of serotonin-like immunoreactive fibres is seen in the ventrolateral wall of the pharynx in our whole-mount preparations of the digestive tract (Figure 7A and Figure S9). The second stomatogastric complex is associated with the midgut. It consists of numerous somata of bipolar neurons that are scattered in a random fashion in the gut wall and send off fibres towards its outer surface, where additional longitudinal fibres are seen (Figure 7B, E and Figure S9). In contrast to the pharynx, no prominent nerves are found within the midgut tissue. The two stomatogastric complexes, i.e., the pharyngeal medullary nerve and the scattered neurons in the midgut wall, do not appear to be connected to each other since they are separated by the tube-like oesophagus, which possesses no immunoreactive neurons (Figures 7A, B, D, 8A–C and Figure S9). Notably, in contrast to the tardigrades, neither anti-serotonin-like immunoreactivity nor anti-acetylated α-tubulin immunolabelling provide evidence for the existence of a stomatogastric ganglion in the onychophoran *Euperipatoides rowelli*.

## Discussion

### No “suboesophageal ganglion” and a dorsal rather than collar-shaped/cycloneuralian brain in tardigrades

Our study revealed distinct patterns of serotonin-like and RFamide-like immunoreactivity in the brain and in the four trunk ganglia of the tardigrade Macrobiotus cf. *harmsworthi* but no staining in the region where a “suboesophageal” or “subpharyngeal ganglion” has been reported [10]–[13]. Furthermore, nuclear staining and anti-tubulin immunolabelling displayed neither a specialised condensation of cells nor neuropils characteristic of ganglia in this region. Instead, all RFamide-like and serotonin-like immunoreactive neurons and the majority of α-tubulin immunoreactive fibres within the cephalic region occur in the tissue above the buccal tube. Therefore, none of the markers used revealed the existence of a “suboesophageal ganglion” in the nervous system of Macrobiotus cf. *harmsworthi*.

Irrespective of the species studied the evidence for the existence of a “suboesophageal”/“subpharyngeal ganglion” in tardigrades is rather weak. Most studies mentioning this structure show only drawings (e.g. in *Macrobiotus hufelandi* [11] and *Styraconyx nanoqsunguak* [13]). Only one study, of the tardigrade *Halobiotus crispae*, claims to have actually stained it [10]. However, the specimens shown were in a contracted condition so that the structure designated as the “subpharyngeal ganglion” could, in our opinion, not be discerned unequivocally as a unit structure against the comparatively high background staining (see fig. 7A, B in ref. [10]). On the other hand, our findings correspond to the observation in *Macrobiotus hufelandi* [9]. Moreover, Hejnol and Schnabel [43] found no anlage of this ganglion in the embryo of the tardigrade *Thulinia stephaniae* using 4D microscopy. We suggest that the structure described previously by some workers as a “suboesophageal” or “subpharyngeal ganglion” [10]–[13] in tardigrades may correspond to either a ventrolateral extension of the brain on each side of the head (see illustrations of the tardigrade nervous system in lateral view in ref. [11]), or a complex of sensory cone ganglia occurring in some tardigrade species [8], [15], [44]. In conclusion, there is no definitive evidence for the existence of a “suboesophageal” or “subpharyngeal ganglion” in Tardigrada and our data from *Macrobiotus* cf. *harmsworthi* support the notion that it is lacking in this group.

Our data further revealed the presence of a prominent anterior ring nerve in front of the dorsally positioned brain, which innervates the mouth lamellae in the tardigrade Macrobiotus cf. *harmsworthi*. Depending on the contraction state of specimens, the ring nerve and the brain appear as either two separate structures or a conglomerate. Apparently, this difference between extended and contracted specimens has not been realised by previous workers, which might have led to different and contradicting interpretations of the tardigrade brain [8]–[10]. According to our findings, the tardigrade ring nerve does not correspond to the cycloneuralian brain [1], as it does not consist of collar-shaped anterior and posterior layers of perikarya separated by a ring-shaped neuropil. Therefore, the nerve ring should not be regarded as part of the tardigrade brain, as it is neither composed of a cluster of neurons, nor does it represent the most prominent anterior condensation of neurons characteristic of animal brains [45]. We suggest that the nerve ring is a derived feature of Tardigrada, as it innervates the peribuccal lamellae surrounding the mouth, which are not found in any other animal group. In contrast, the bilaterally symmetric, dorsal brain of Macrobiotus cf. *harmsworthi* is comparable to the brain of other protostomes, including the onychophorans and arthropods [4], [45], and might thus represent an ancestral feature of Panarthropoda.

### The stomatogastric ganglion: a potential synapomorphy of Tardigrada and Arthropoda

**Figure 9:**
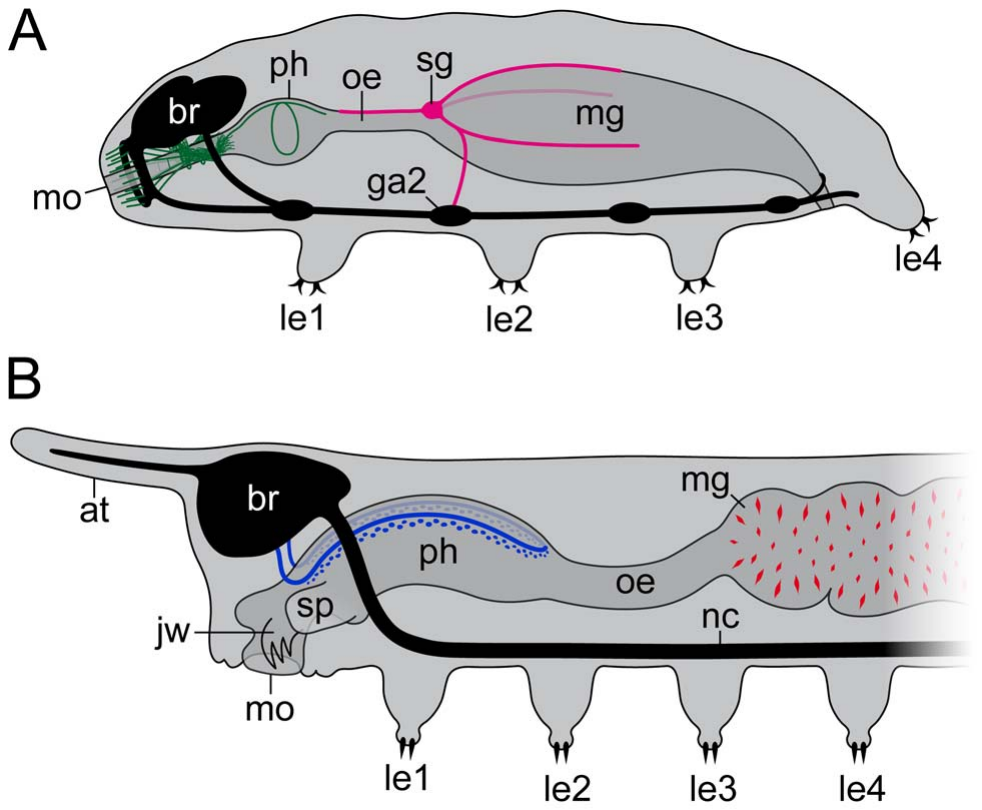
Diagrams summarising the innervation pattern of foregut and midgut structures in Tardigrada and Onychophora. (**A**) Stomodeal complex innervating the mouth, the stylet and the pharynx (green) and the stomatogastric ganglion with its projections (magenta) in the tardigrade *Macrobiotus* cf. *harmsworthi.* (**B**) In the onychophoran *Euperipatoides rowelli*, the ectodermal pharynx is innervated by a loop-like, medullary pharyngeal nerve (blue), which is accompanied by neuronal somata. The endodermal midgut shows numerous neuronal cell bodies scattered in the gut wall (red). Abbreviations: at, antenna; br, brain; ga2, second trunk ganglion; jw, jaw; le1–le4, walking legs 1 to 4; mg, midgut; mo, mouth; nc, nerve cord; oe, oesophagus; ph, pharynx; sg, stomatogastric ganglion; sp, slime papilla.

In this study, we identified two hitherto unknown components of the stomatogastric nervous system in Macrobiotus cf. *harmsworthi*, which supply the midgut and foregut structures in this species. The first component comprises the stomodeal complex consisting of fibres innervating the anterior mouth lamellae, two bundles of neurites originating from the brain and running posteriorly to supply the stylet musculature, and the pharyngeal nerve (green in Figure 9A). This stomodeal complex might correspond to the pharyngeal nerve (“Nervus stomodealis” [46]) of onychophorans (blue in Figure 9B) since both neural structures connect to the brain and innervate portions of the ectodermal foregut, including the pharynx. However, the onychophoran pharyngeal nerve is accompanied by serotonin-like immunoreactive neuronal somata and, therefore, shows a medullary organisation, which is not evident in tardigrades. Due to these differences, the homology of nerves and fibres supplying the foregut structures in tardigrades and onychophorans is uncertain. Likewise, because of apparent structural and positional differences, the homology of the corresponding nerves innervating different foregut structures in various arthropods [47]–[49] remains open for discussion.

The second major component of the tardigrade stomatogastric nervous system, described here for the first time, comprises a clearly defined ganglion containing serotonin-like immunoreactive neurons that supply the ectodermal oesophagus and the endodermal midgut (magenta in Figure 9A). In contrast to this innervation pattern, our data from Onychophora show numerous single, bipolar neurons scattered in a random fashion throughout the midgut wall (red in Figure 9B). Accordingly, the stomatogastric nervous system in Onychophora cannot be assigned to a specific segment. Our finding is in line with a previous description [23] of numerous serotonergic somata that give rise to a plexus of nerve fibres on the surface of the gut in the onychophoran Metaperipatus blainvillei. The distributed pattern of midgut innervation identified in Onychophora might be an ancestral feature (plesiomorphy) of Panarthropoda since it is also found in various other protostomes [50].

In contrast, arthropods show an innervation pattern, which is similar to that in tardigrades, as they also have stomatogastric ganglia (termed also “frontal”, “rostral” or “stomodeal ganglia”) [16]–[19], [49], [51], [52]. The most striking similarity is seen between tardigrades, and crustaceans and insects since representatives of these two arthropod groups typically possess an unpaired stomatogastric ganglion associated with the dorsal wall of the foregut [16], [53]–[56]. Regarding insects, it is important to stress that only the frontal ganglion is relevant since the hypocerebral ganglion has no direct connection to the central nervous system (review [53]) and its evolutionary history and homology is unclear. In chelicerates and myriapods, including xiphosurans [57], [58] and symphylans [17], the stomatogastric ganglia originate as paired outgrowths from the walls of the stomodaeum. During embryogenesis, the paired ganglionic cell masses, which are linked by a stomodeal bridge, fuse with the brain tissue but retain a ventrolateral position beneath the oesophagus [17], [57], [59]. Irrespective of whether the stomatogastric ganglion anlagen are paired or unpaired, arthropods and tardigrades share a centralised stomatogastric nervous system, which contrasts with the distributed innervation pattern in onychophorans and other protostomes [23], [50].

We suggest that the stomatogastric ganglia of tardigrades and arthropods are homologous because they represent accumulations of neurons that innervate corresponding structures: the ectodermal foregut and the endodermal midgut and connect to the central nervous system. Whether the stomatogastric ganglion has been lost in onychophorans or whether it has evolved in the tardigrade/arthropod lineage after the divergence of onychophorans depends on the phylogenetic position of tardigrades, which is still controversial. Currently, Tardigrada is regarded as either the sister group to Arthropoda, to Onychophora, to Onychophora + Arthropoda, or to one of the cycloneuralian taxa, such as Nematoda (e.g. [60]–[69]). Since onychophorans lack a stomatogastric ganglion and show a distributed innervation pattern of the gut – a condition which is also found in various outgroups [23], [50] – our data suggest that the stomatogastric ganglion is a potential synapomorphy supporting the sister group relationship of Tardigrada and Arthropoda.

### Implications for head segmentation in tardigrades

**Figure 10:**
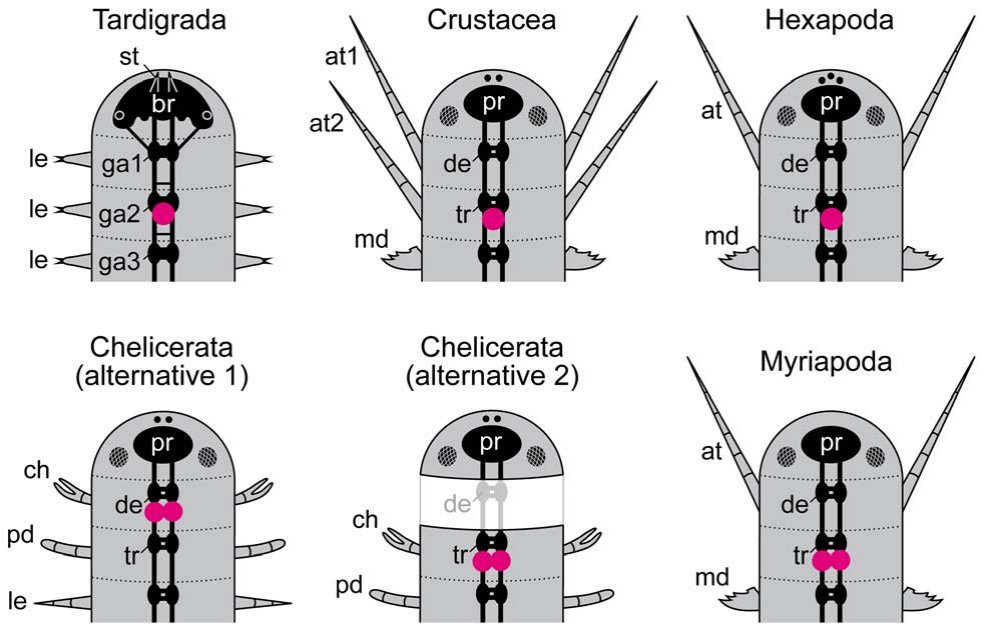
Diagram showing the alignment of head segments and segmental regions of the nervous system in tardigrades and arthropods. Circles filled with magenta indicate the position of the stomatogastric ganglia (often referred to as the “frontal”, “rostral” or “stomodeal ganglia” in some groups). Note that the protocerebrum, deutocerebrum and tritocerebrum do not form separate ganglia but are usually fused to a syncerebrum [45] in adult arthropods. Note also the two alternative alignments for chelicerates, consistent with either the expression pattern of Hox genes [6], [70], [71] (alternative 1) or with the anterior border and periodicity of expression of the pair-rule genes runt and paired [49], [72] (alternative 2). According to the second alternative, the deutocerebral segment has been reduced in chelicerates (marked by white colour and light-grey lines). Small circles in the ocular segment indicate the position of eyes in tardigrades and median ocelli in arthropods. Hatched ovals illustrate the position of compound eyes in arthropods. Abbreviations: at, antenna; at1 and at2, first and second antennae; ch, chelicera; de, deutocerebrum; ga1–ga3, trunk ganglia 1 to 3; le, walking leg; md, mandible; mx, maxilla; pd, pedipalp; pr, protocerebrum; st, stylet; tr, tritocerebrum.

The stomatogastric ganglion of tardigrades is associated with the second trunk ganglion, which is located in the second leg-bearing segment. This finding is of major significance since the position of the stomatogastric ganglia is conserved among different arthropod groups. In myriapods, crustaceans and hexapods, these ganglia are associated with the third/tritocerebral body segment [5],[16]–[19], although in chelicerates, where the situation is less clear, there are two alternative alignments of head segments (Figure 10). According to the first alternative, which is based on the expression pattern of the anterior Hox genes labial, proboscipedia and Deformed, the cheliceral segment of chelicerates is homologous to the second body segment in other arthropods [6], [70], [71]. However, this hypothesis (alternative 1 in Figure 10) requires the assumption of an anterior shift in position of stomatogastric ganglia in chelicerates [58]. The second hypothesis (alternative 2 in Figure 10) is based on the association of stomatogastric ganglia with cheliceral neuromeres [49]. This hypothesis contradicts the alignment of head segments based on Hox gene expression data but receives support from the expression pattern of the pair-rule genes runt and paired in a mite, which shows a specific anterior boundary and periodicity [72] (discussed in ref. [49]). According to this hypothesis, the position of stomatogastric ganglia in chelicerates corresponds to the conserved position of these structures in other arthropods. However, this necessitates the assumption that the second/deutocerebral body segment was lost in chelicerates [49], [52].

Irrespective of the controversy surrounding the alignment of head segments in chelicerates, the position of the stomatogastric ganglion in tardigrades can be used as a landmark for clarifying the homology of anterior body segments in tardigrades and arthropods, provided the stomatogastric ganglia are truly homologous. According to the position of the stomatogastric ganglion, the second trunk segment of tardigrades is homologous to the third/tritocerebral segment of myriapods, crustaceans and hexapods (Figure 10). This leaves only room for two segmental units of the nervous system in front of the second trunk ganglion in tardigrades, i.e., the first trunk ganglion, and the brain. The inferred number of two segmental units is supported by the lack of a “suboesophageal ganglion”, indicating that there are no additional segments in the tardigrade head. Moreover, an expression study of Pax3/7 and Engrailed proteins in a tardigrade revealed only two expression domains in front of the second leg-bearing segment [73]. This suggests that the tardigrade brain is a homologue of the arthropod protocerebrum, whereas the first trunk ganglion is homologous to the arthropod deutocerebrum.

Taken together, we propose that the tardigrade head consists of only one, protocerebral segment, followed by the trunk composed of four leg-bearing segments. The protocerebral segment is associated with the stylet apparatus, which might be a derivative of the anterior-most pair of appendages [74], corresponding to the antennae of extant onychophorans and Cambrian lobopodians [3]–[5], [75]–[78]. Although conjectural, the validity of this proposed alignment of head segments between tardigrades and other panarthropods can be readily tested in future studies by analysing the expression patterns of the anterior Hox genes in tardigrades. According to our alignment, we predict that the anterior boundaries of the expression domain of labial in the tardigrade embryo will lie between the first and second leg-bearing segments, while that of Deformed will occur between the second and third leg-bearing segments.

## Supporting Information

Figure S1 **Organisation of the nervous system in the tardigrade Macrobiotus cf.** ***harmsworthi*** **.** Version for the colour-blind. RFamide-like immunoreactivity (magenta) and DNA labelling (green). Maximum projections of confocal z-series. Anterior is up in all images except for B in which anterior is left. Note that no “suboesophageal” or “subpharyngeal ganglion” is evident in its presumptive position (asterisks in A–C). Arrowheads in A–C point to varicose swellings in the core of each trunk ganglion. Arrows in B and D indicate two anterior, extra-cerebral RFamide-like immunoreactive cell bodies. (**A**) Specimen in ventral view showing four trunk ganglia linked by somata-free connectives. (**B**) Specimen in lateral view revealing the dorsal position of the brain (dotted line). (**C**) Anterior end in ventral view. (**D**) Anterior end in dorsal view with details of central brain neuropil. Dotted line indicates the shape of the brain with its lobes. Abbreviations: br, brain; cn, connectives; ga1–ga4, trunk ganglia 1 to 4; il, inner brain lobe; le1–le4, walking legs 1 to 4; np, central brain neuropil; oc, outer connective; ol, outer brain lobe; ml, median brain lobe; so, neuronal somata. Scale bars: 25 µm (A), and 10 µm (B–D).
(TIF)

Figure S2 **Organisation of the nervous system in the tardigrade Macrobiotus cf.** ***harmsworthi*** **.** RFamide-like immunoreactive staining. Maximum projections of confocal z-series. Anterior is up in all images except for B in which anterior is left. Note that no “suboesophageal” or “subpharyngeal ganglion” is evident in its presumptive position (asterisks in A–C). Arrowheads in A–C point to varicose swellings in the core of each trunk ganglion. Arrows in B and D indicate two anterior, extra-cerebral RFamide-like immunoreactive cell bodies. (**A**) Specimen in ventral view showing four trunk ganglia linked by somata-free connectives. (**B**) Specimen in lateral view revealing the dorsal position of the brain (dotted line). (**C**) Anterior end in ventral view. (**D**) Anterior end in dorsal view with details of central brain neuropil. Dotted line indicates the shape of the brain with its lobes. Abbreviations: br, brain; cn, connectives; ga1–ga4, trunk ganglia 1 to 4; il, inner brain lobe; le1–le4, walking legs 1 to 4; np, central brain neuropil; oc, outer connective; ol, outer brain lobe; ml, median brain lobe; so, neuronal somata. Scale bars: 25 µm (A), and 10 µm (B–D).
(TIF)

Figure S3 **Characteristics of the ventral nervous system in the tardigrade Macrobiotus cf.** ***harmsworthi*** **.** Combined anti-tyrosinated and anti-acetylated α-tubulin immunolabelling. Projections of confocal z-series. Anterior is up. (**A**) Overview of a portion of the ventral nervous system showing the fibre masses within the third and the fourth trunk ganglia. The ganglia are linked by somata-free connectives along the antero-posterior body axis. Numerous contralateral projections and commissures are seen within each ganglion. Arrows point to additional commissures, which link the two connectives in front of each trunk ganglion. (**B**) Detail of the second trunk ganglion showing contralateral projections and commissures (arrowheads) in the periphery and within the central fibre mass of the ganglion. Arrow points to an additional commissure in front of the second trunk ganglion. Abbreviations: cn, connective; ga3, third trunk ganglion; ga4, fourth trunk ganglion; ln, leg nerves; pn, peripheral nerve. Scale bars: 10 µm (A), and 5 µm (B).
(TIF)

Figure S4 **Anterior, extra-cerebral, serotonin-like immunoreactive cells in the tardigrade Macrobiotus cf.** ***harmsworthi*** **.** Confocal micrographs. Anterior is up. (**A–D**) Selected optical sections from a confocal z-series. Note the cell bodies of ten serotonin-like immunoreactive neurons (numbered) associated with the mouth cone, which also shows a strong signal. Abbreviations: mc, mouth cone; so, neuronal somata within the brain. Scale bars: 10 µm (A–D).
(TIF)

Figure S5 **Neural structures in the anterior end of an extended specimen of Macrobiotus cf.** ***harmsworthi*** **.** Combined anti-tyrosinated and anti-acetylated α-tubulin immunolabelling. Depth-coded maximum projection of a confocal z-series. Anterior is up. Arrowheads point to the nerve ring, which gives rise to numerous anterior neurites innervating the peribuccal/mouth lamellae. Abbreviations: as, anterolateral sensory fields; cn, connective; co, commissure of the stomodeal complex (cf. Figure 5C); ga1, first trunk ganglion; ln, leg nerves; ne, neurites supplying the peribuccal/mouth lamellae; oc, outer connective; pn, peripheral nerve; ps, posterolateral sensory fields. Scale bar: 30 µm.
(TIF)

Figure S6 **Innervation of stylet and pharynx in the tardigrade Macrobiotus cf.** ***harmsworthi*** **.** Projections of subsets of optical sections from confocal z-series. Anterior is up. Arrows point to fibres supplying the pharynx. (**A**) Combined anti-tyrosinated and anti-acetylated α-tubulin immunolabelling. Arrowheads point to neurite bundles innervating the stylet musculature. (**B**) Serotonin-like immunoreactivity. Autofluorescent structures, including the calcified elements of the stylet, are shown in blue. Abbreviations: as, anterolateral sensory field; en, terminal of the anterior neurite originating from the stomatogastric ganglion and running along the oesophagus; ex, extra-cerebral neuronal somata; mc, mouth cone; mp, macroplacoids; ne, neurites supplying the peribuccal/mouth lamellae; oc, outer connective; pn, peripheral nerve; ps, posterolateral sensory fields. so, neuronal somata within the brain; st, stylet; un, unpaired posterior nerve/neurite. Scale bars: 10 µm (A, B).
(TIF)

Figure S7 **Comparison of anterior neural structures in an extended (left half) and a contracted specimen (right half) of the tardigrade Macrobiotus cf.** ***harmsworthi*** **.** Maximum projections of confocal z-series. Equivalent structures are highlighted by corresponding artificial colours. Arrowheads point to the nerve ring, which gives rise to neurites innervating the peribuccal/mouth lamellae. Abbreviations: as, anterolateral sensory fields; bd, neurite bundles innervating the stylet musculature; cn, connective; np, central brain neuropil; oc, outer connective; ps, posterolateral sensory fields. Scale bars: 20 µm.
(TIF)

Figure S8 **Anterior neural structures and oesophageal neurite in the tardigrade Macrobiotus cf.** ***harmsworthi*** **.** Combined anti-tyrosinated and anti-acetylated α-tubulin immunolabelling of a contracted specimen. Projection of a confocal z-series. Anterior is up. Arrows indicate neurite bundles innervating the stylet musculature. Arrowhead points to the nerve ring, which gives rise to neurites innervating the peribuccal/mouth lamellae. The shape of the pharynx is indicated by dotted lines. Note that the anterior oesophageal neurite terminates posterior to the pharynx. Abbreviations: as, anterolateral sensory fields; en, oesophageal neurite; ne, neurites supplying the peribuccal/mouth lamellae; np, central brain neuropil; ph, pharynx; pn, peripheral nerve; ps, posterolateral sensory fields; st, stylet. Scale bar: 20 µm.
(TIF)

Figure S9 **Innervation pattern of pharynx, oesophagus and midgut in the onychophoran Euperipatoides rowelli.** Version for the colour-blind. Confocal micrographs. (**A, B**) Anti-serotonin immunolabelling of a dissected digestive tract. Anterior is left. (**C–E**) Triple-labelled vibratome cross-sections using an anti-acetylated α-tubulin antibody (green), phalloidin-rhodamine (magenta), and the DNA-selective dye Hoechst (blue). Dorsal is up. (**A**) Whole-mount preparation showing the pharyngeal loop nerve (lo). Inset, which is a projection of only a few focal planes, illustrates numerous serotonin-like immunoreactive somata associated with this nerve (arrowheads). (**B**) Detail of transition region between oesophagus and midgut in the same whole-mount preparation as in A. Note the scattered arrangement of neuronal cell bodies within the gut wall (arrowheads). Inset, which is a projection of only a few focal planes, shows a nerve fibre and details of serotonin-like immunoreactive somata of bipolar neurons within the gut wall (arrowheads). (**A–E**) Vibratome cross-sections of the pharynx (ph), oesophagus (oe) and midgut (mg) showing their innervation pattern. Arrows in C point to fibres supplying the pharyngeal musculature. Arrowheads in E indicate single neurons in the wall of the gut. Note the lack of nerves and neuronal cell bodies in the oesophagus wall in D. Abbreviations: bf, bundle of fibres in the pharyngeal wall; fi, serotonin-like immunoreactive fibre on the outer surface of the gut wall; lo, pharyngeal loop nerve; mg, midgut; nc, nerve cord; oe, oesophagus; ph, pharynx; sl, slime glands. Scale bars: 100 µm (A–E), and 25 µm (insets in A and B).
(TIF)

Movie S1 **Details of the brain in the tardigrade Macrobiotus cf.** ***harmsworthi*** **.** Movie based on a confocal z-series. RFamide-like immunoreactivity. Anterior is up.
(MP4)

Movie S2 **Details of the brain in the tardigrade Macrobiotus cf.** ***harmsworthi*** **.** Movie based on a confocal z-series. Serotonin-like (green) and anti-acetylated α-tubulin immunoreactivity showing the anterolateral and posterolateral sensory fields (red). Anterior is up.
(MP4)

Movie S3 **Details of the anterior neural and sensory structures in the tardigrade Macrobiotus cf.** ***harmsworthi*** **.** Movie based on a confocal z-series. Combined anti-tyrosinated and anti-acetylated α-tubulin immunoreactivity. Anterior is up.
(MP4)
