# Supplementary figures and images for: Neural Markers Reveal a One-Segmented Head in Tardigrades (Water Bears)
Source: PLoS One. 2013 Mar 13;8(3):e59090. doi: 10.1371/journal.pone.0059090 (PMC3596308; doi:10.1371/journal.pone.0059090)

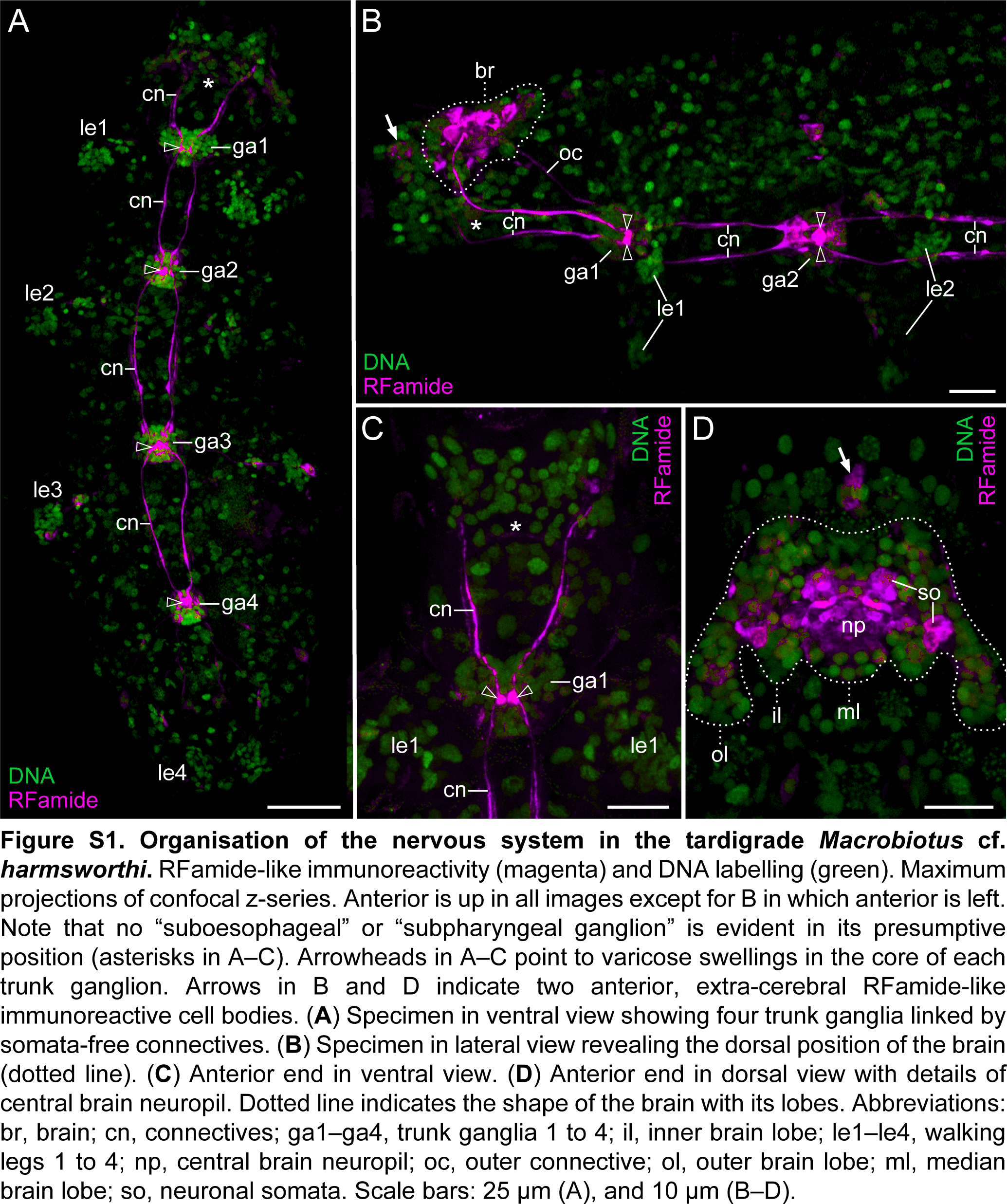

Supplement: Figure S1 — Organisation of the nervous system in the tardigrade Macrobiotus cf. harmsworthi . Version for the colour-blind. RFamide-like immunoreactivity (magenta) and DNA labelling (green). Maximum projections of confocal z-series. Anterior is up in all images except for B in which anterior is left. Note that no “suboesophageal” or “subpharyngeal ganglion” is evident in its presumptive position (asterisks in A–C). Arrowheads in A–C point to varicose swellings in the core of each trunk ganglion. Arrows in B and D indicate two anterior, extra-cerebral RFamide-like immunoreactive cell bodies. (A) Specimen in ventral view showing four trunk ganglia linked by somata-free connectives. (B) Specimen in lateral view revealing the dorsal position of the brain (dotted line). (C) Anterior end in ventral view. (D) Anterior end in dorsal view with details of central brain neuropil. Dotted line indicates the shape of the brain with its lobes. Abbreviations: br, brain; cn, connectives; ga1–ga4, trunk ganglia 1 to 4; il, inner brain lobe; le1–le4, walking legs 1 to 4; np, central brain neuropil; oc, outer connective; ol, outer brain lobe; ml, median brain lobe; so, neuronal somata. Scale bars: 25 µm (A), and 10 µm (B–D). (TIF) [file pone.0059090.s001.tif]

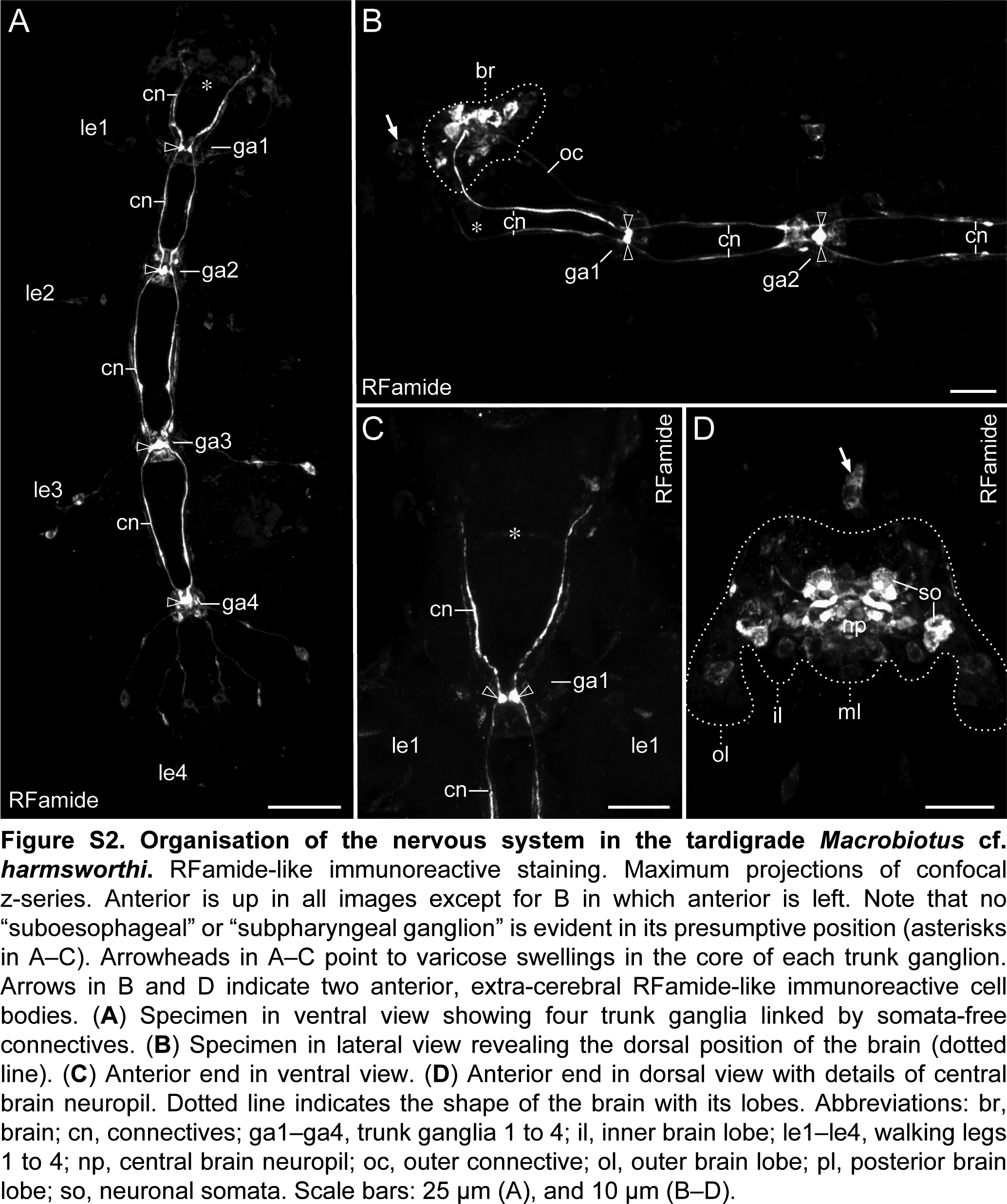

Supplement: Figure S2 — Organisation of the nervous system in the tardigrade Macrobiotus cf. harmsworthi . RFamide-like immunoreactive staining. Maximum projections of confocal z-series. Anterior is up in all images except for B in which anterior is left. Note that no “suboesophageal” or “subpharyngeal ganglion” is evident in its presumptive position (asterisks in A–C). Arrowheads in A–C point to varicose swellings in the core of each trunk ganglion. Arrows in B and D indicate two anterior, extra-cerebral RFamide-like immunoreactive cell bodies. (A) Specimen in ventral view showing four trunk ganglia linked by somata-free connectives. (B) Specimen in lateral view revealing the dorsal position of the brain (dotted line). (C) Anterior end in ventral view. (D) Anterior end in dorsal view with details of central brain neuropil. Dotted line indicates the shape of the brain with its lobes. Abbreviations: br, brain; cn, connectives; ga1–ga4, trunk ganglia 1 to 4; il, inner brain lobe; le1–le4, walking legs 1 to 4; np, central brain neuropil; oc, outer connective; ol, outer brain lobe; ml, median brain lobe; so, neuronal somata. Scale bars: 25 µm (A), and 10 µm (B–D). (TIF) [file pone.0059090.s002.tif]

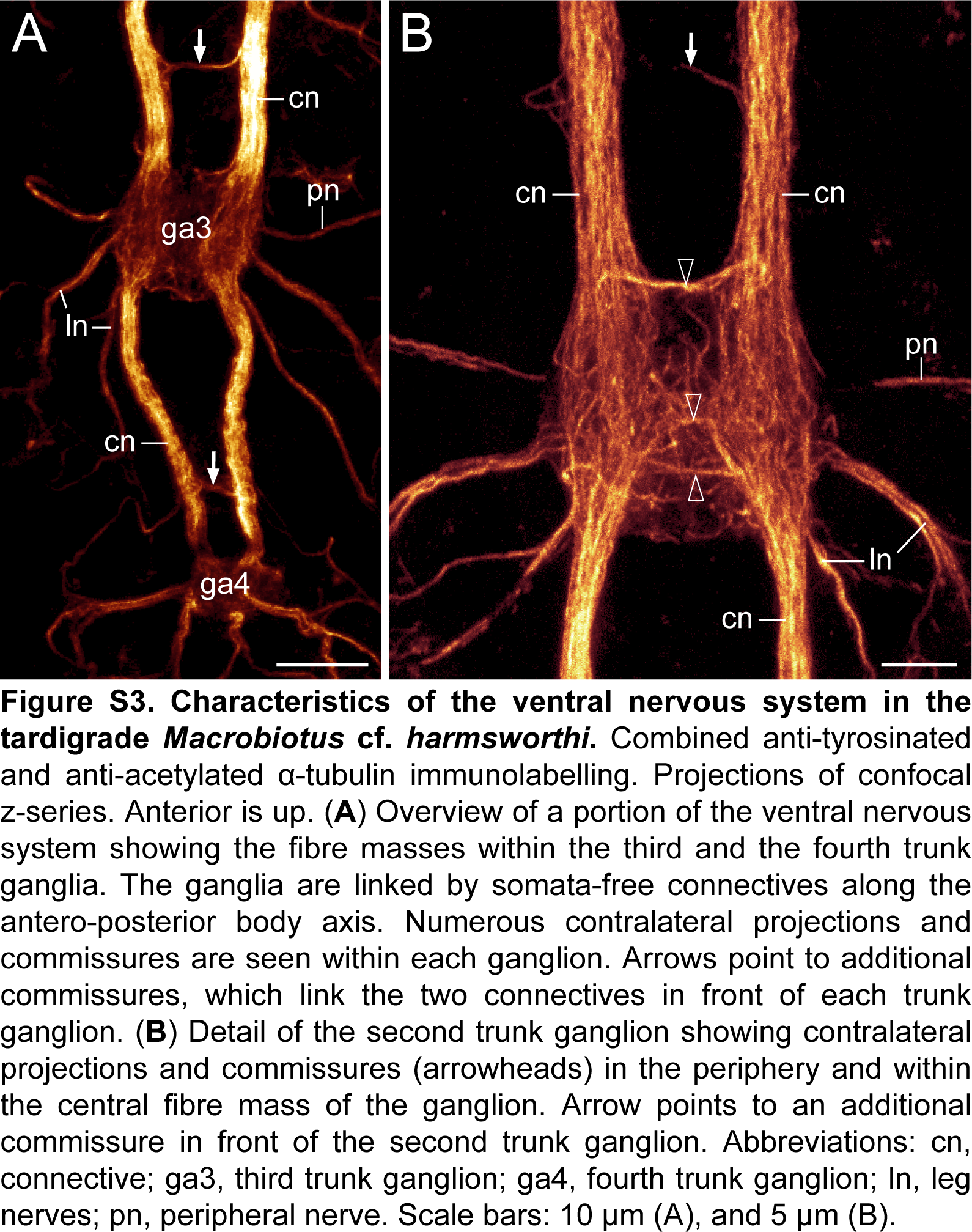

Supplement: Figure S3 — Characteristics of the ventral nervous system in the tardigrade Macrobiotus cf. harmsworthi . Combined anti-tyrosinated and anti-acetylated α-tubulin immunolabelling. Projections of confocal z-series. Anterior is up. (A) Overview of a portion of the ventral nervous system showing the fibre masses within the third and the fourth trunk ganglia. The ganglia are linked by somata-free connectives along the antero-posterior body axis. Numerous contralateral projections and commissures are seen within each ganglion. Arrows point to additional commissures, which link the two connectives in front of each trunk ganglion. (B) Detail of the second trunk ganglion showing contralateral projections and commissures (arrowheads) in the periphery and within the central fibre mass of the ganglion. Arrow points to an additional commissure in front of the second trunk ganglion. Abbreviations: cn, connective; ga3, third trunk ganglion; ga4, fourth trunk ganglion; ln, leg nerves; pn, peripheral nerve. Scale bars: 10 µm (A), and 5 µm (B). (TIF) [file pone.0059090.s003.tif]

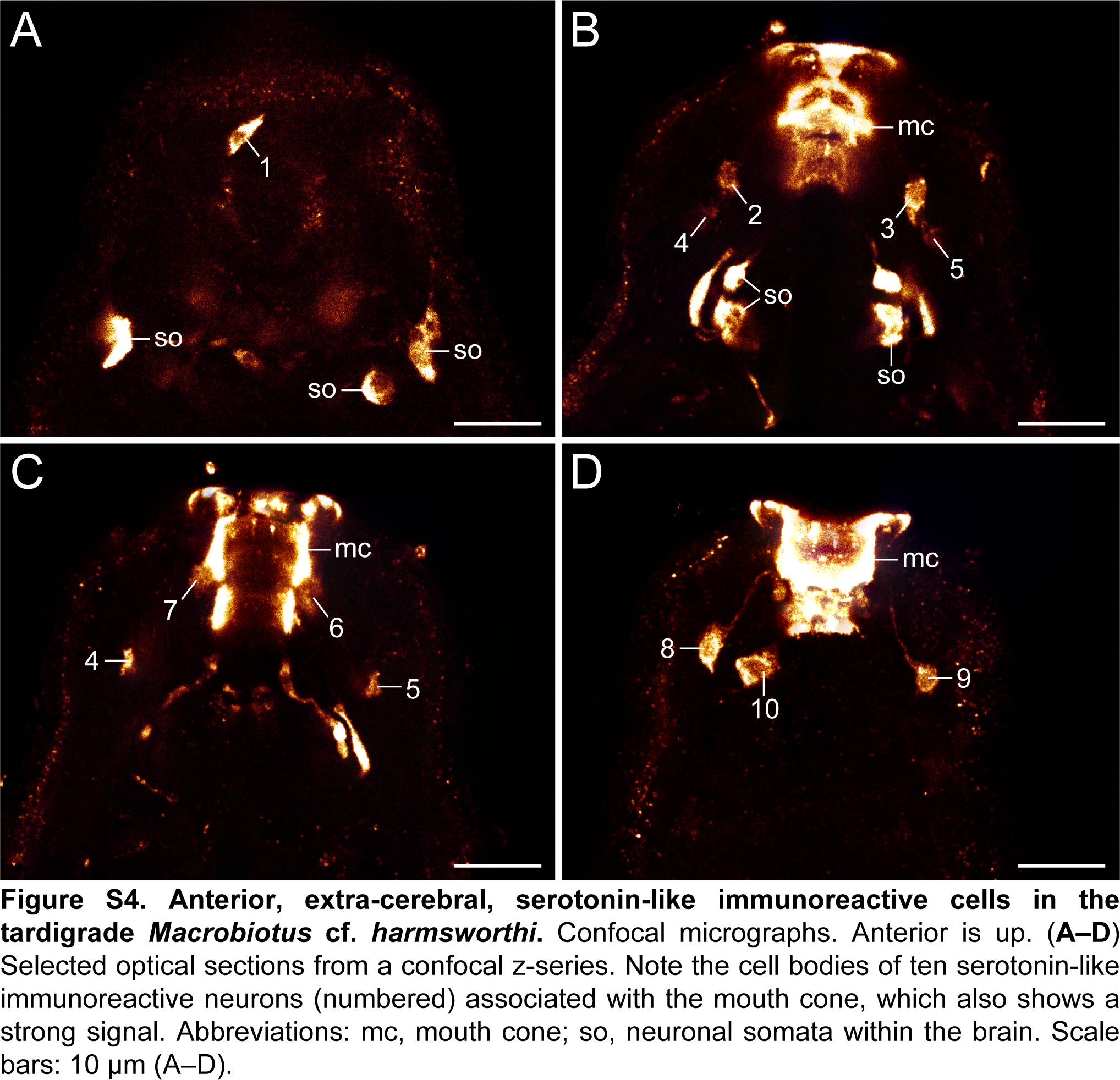

Supplement: Figure S4 — Anterior, extra-cerebral, serotonin-like immunoreactive cells in the tardigrade Macrobiotus cf. harmsworthi . Confocal micrographs. Anterior is up. (A–D) Selected optical sections from a confocal z-series. Note the cell bodies of ten serotonin-like immunoreactive neurons (numbered) associated with the mouth cone, which also shows a strong signal. Abbreviations: mc, mouth cone; so, neuronal somata within the brain. Scale bars: 10 µm (A–D). (TIF) [file pone.0059090.s004.tif]

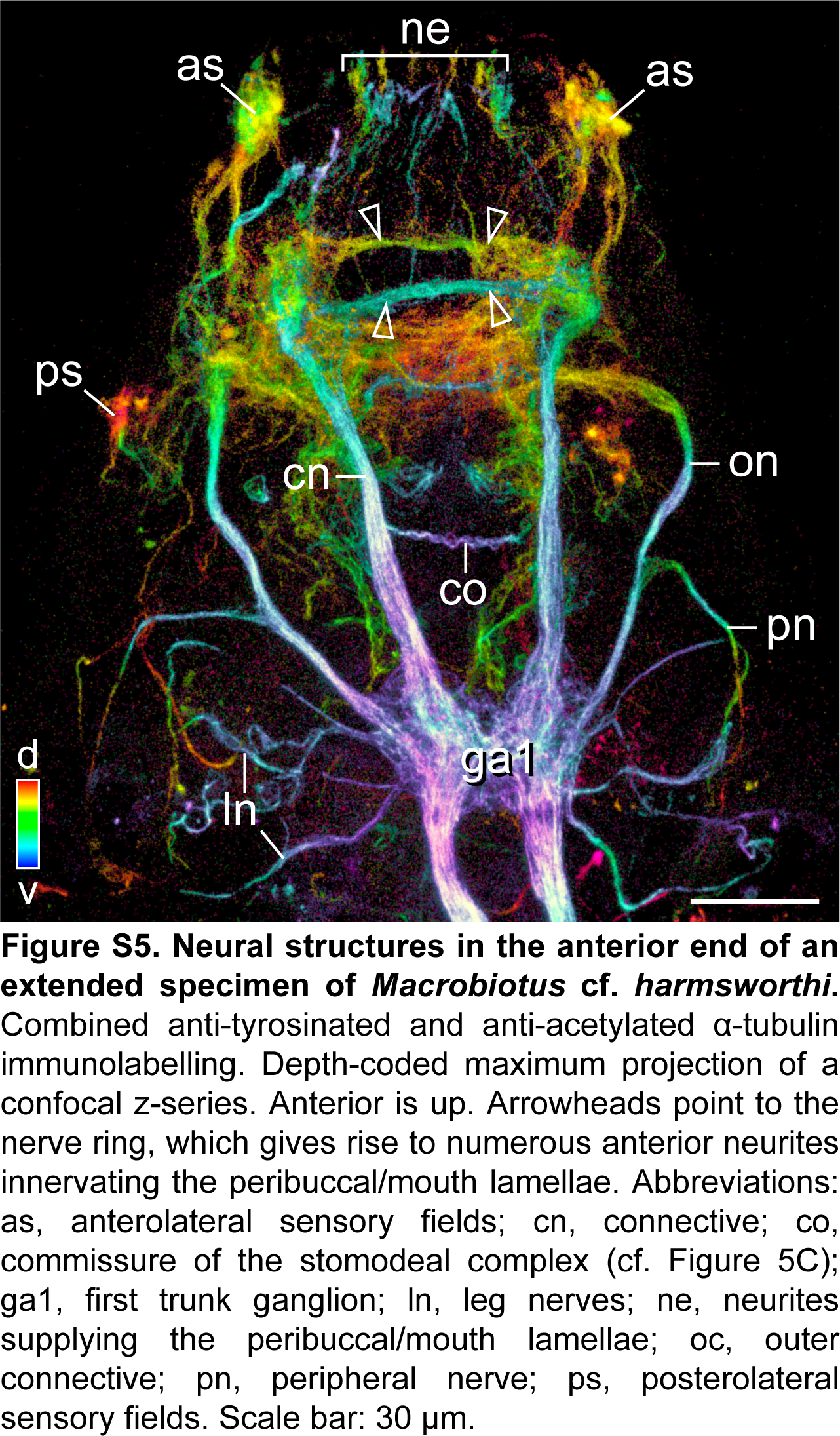

Supplement: Figure S5 — Neural structures in the anterior end of an extended specimen of Macrobiotus cf. harmsworthi . Combined anti-tyrosinated and anti-acetylated α-tubulin immunolabelling. Depth-coded maximum projection of a confocal z-series. Anterior is up. Arrowheads point to the nerve ring, which gives rise to numerous anterior neurites innervating the peribuccal/mouth lamellae. Abbreviations: as, anterolateral sensory fields; cn, connective; co, commissure of the stomodeal complex (cf. Figure 5C); ga1, first trunk ganglion; ln, leg nerves; ne, neurites supplying the peribuccal/mouth lamellae; oc, outer connective; pn, peripheral nerve; ps, posterolateral sensory fields. Scale bar: 30 µm. (TIF) [file pone.0059090.s005.tif]

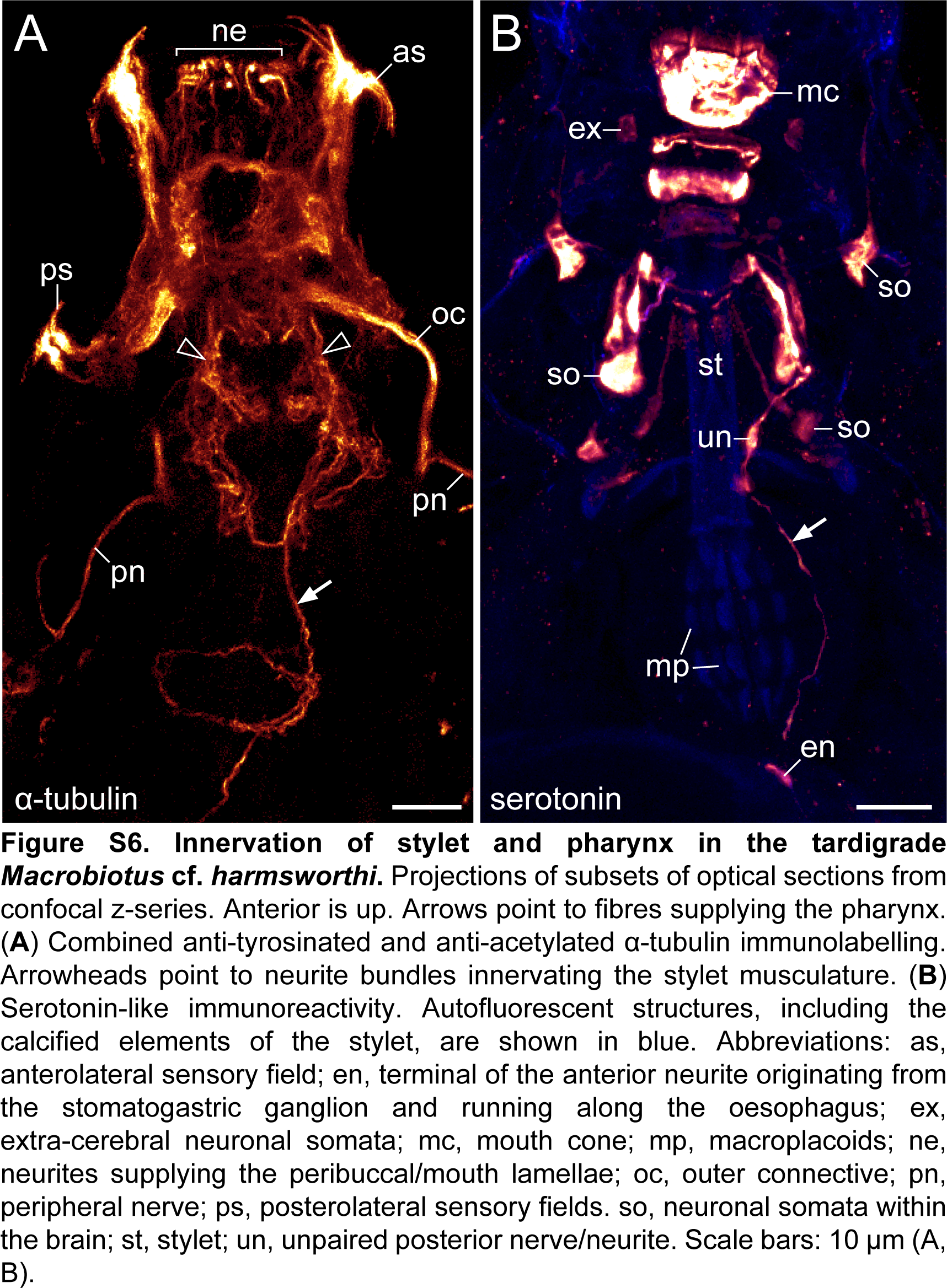

Supplement: Figure S6 — Innervation of stylet and pharynx in the tardigrade Macrobiotus cf. harmsworthi . Projections of subsets of optical sections from confocal z-series. Anterior is up. Arrows point to fibres supplying the pharynx. (A) Combined anti-tyrosinated and anti-acetylated α-tubulin immunolabelling. Arrowheads point to neurite bundles innervating the stylet musculature. (B) Serotonin-like immunoreactivity. Autofluorescent structures, including the calcified elements of the stylet, are shown in blue. Abbreviations: as, anterolateral sensory field; en, terminal of the anterior neurite originating from the stomatogastric ganglion and running along the oesophagus; ex, extra-cerebral neuronal somata; mc, mouth cone; mp, macroplacoids; ne, neurites supplying the peribuccal/mouth lamellae; oc, outer connective; pn, peripheral nerve; ps, posterolateral sensory fields. so, neuronal somata within the brain; st, stylet; un, unpaired posterior nerve/neurite. Scale bars: 10 µm (A, B). (TIF) [file pone.0059090.s006.tif]

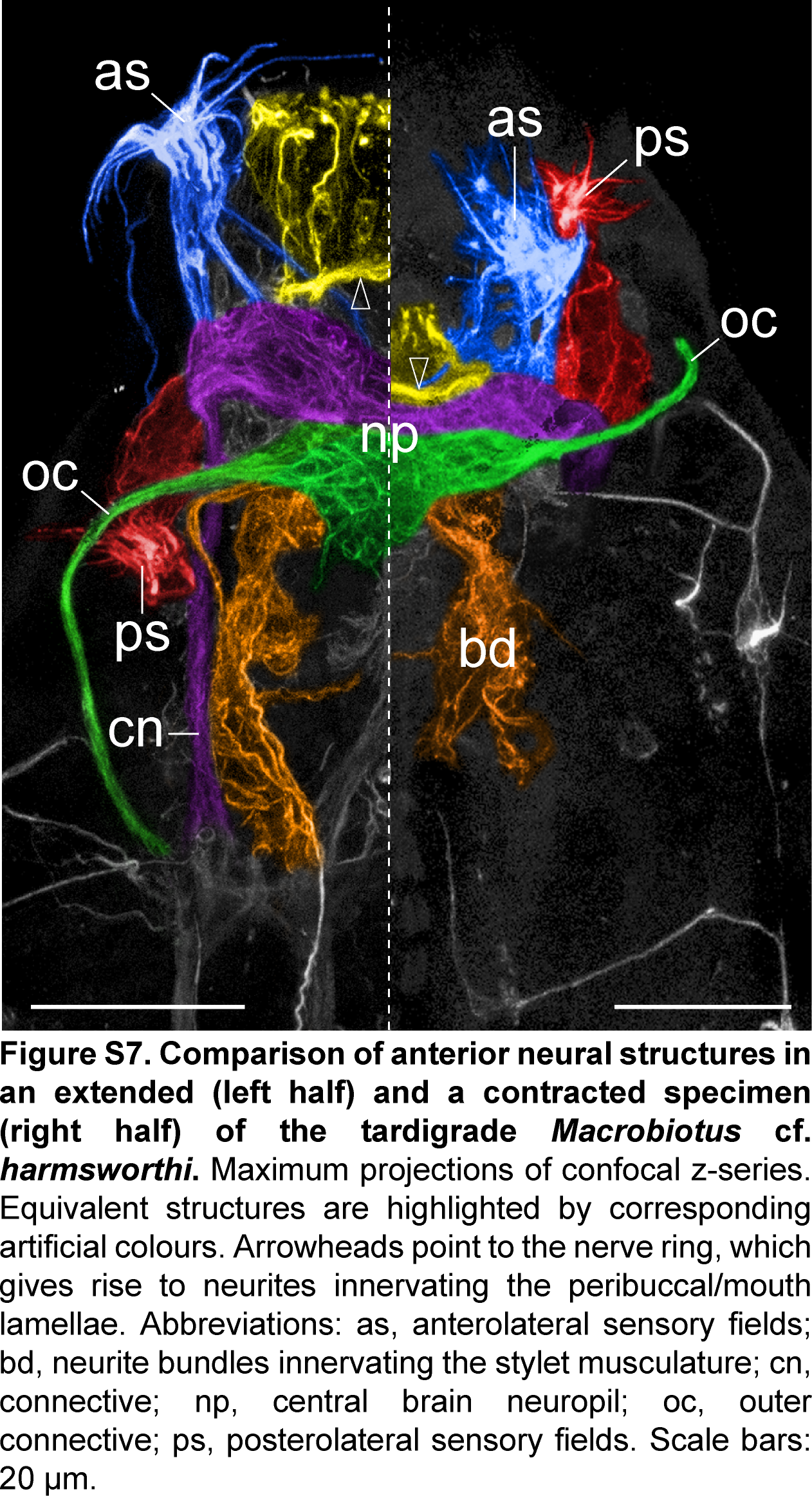

Supplement: Figure S7 — Comparison of anterior neural structures in an extended (left half) and a contracted specimen (right half) of the tardigrade Macrobiotus cf. harmsworthi . Maximum projections of confocal z-series. Equivalent structures are highlighted by corresponding artificial colours. Arrowheads point to the nerve ring, which gives rise to neurites innervating the peribuccal/mouth lamellae. Abbreviations: as, anterolateral sensory fields; bd, neurite bundles innervating the stylet musculature; cn, connective; np, central brain neuropil; oc, outer connective; ps, posterolateral sensory fields. Scale bars: 20 µm. (TIF) [file pone.0059090.s007.tif]

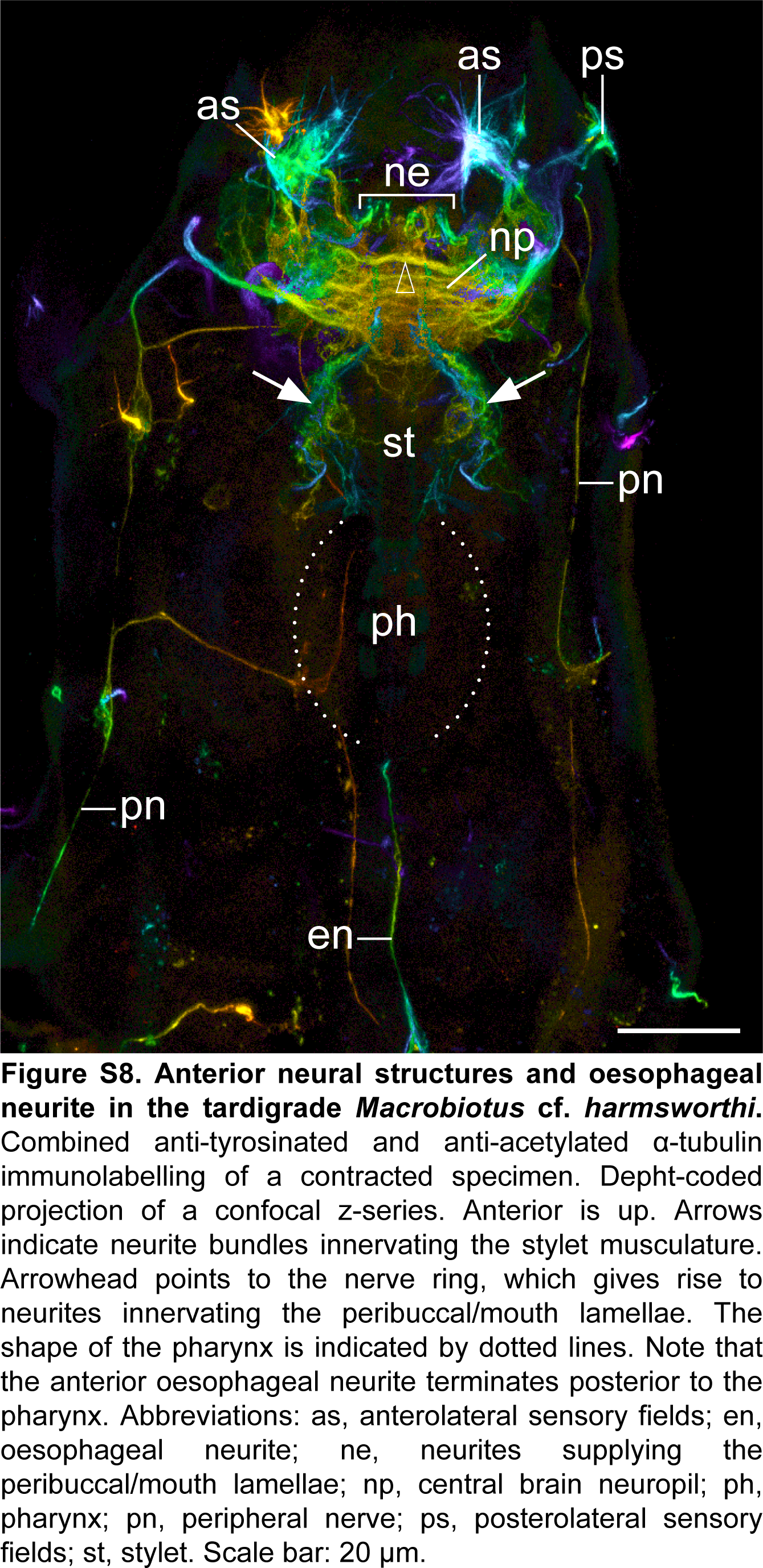

Supplement: Figure S8 — Anterior neural structures and oesophageal neurite in the tardigrade Macrobiotus cf. harmsworthi . Combined anti-tyrosinated and anti-acetylated α-tubulin immunolabelling of a contracted specimen. Projection of a confocal z-series. Anterior is up. Arrows indicate neurite bundles innervating the stylet musculature. Arrowhead points to the nerve ring, which gives rise to neurites innervating the peribuccal/mouth lamellae. The shape of the pharynx is indicated by dotted lines. Note that the anterior oesophageal neurite terminates posterior to the pharynx. Abbreviations: as, anterolateral sensory fields; en, oesophageal neurite; ne, neurites supplying the peribuccal/mouth lamellae; np, central brain neuropil; ph, pharynx; pn, peripheral nerve; ps, posterolateral sensory fields; st, stylet. Scale bar: 20 µm. (TIF) [file pone.0059090.s008.tif]

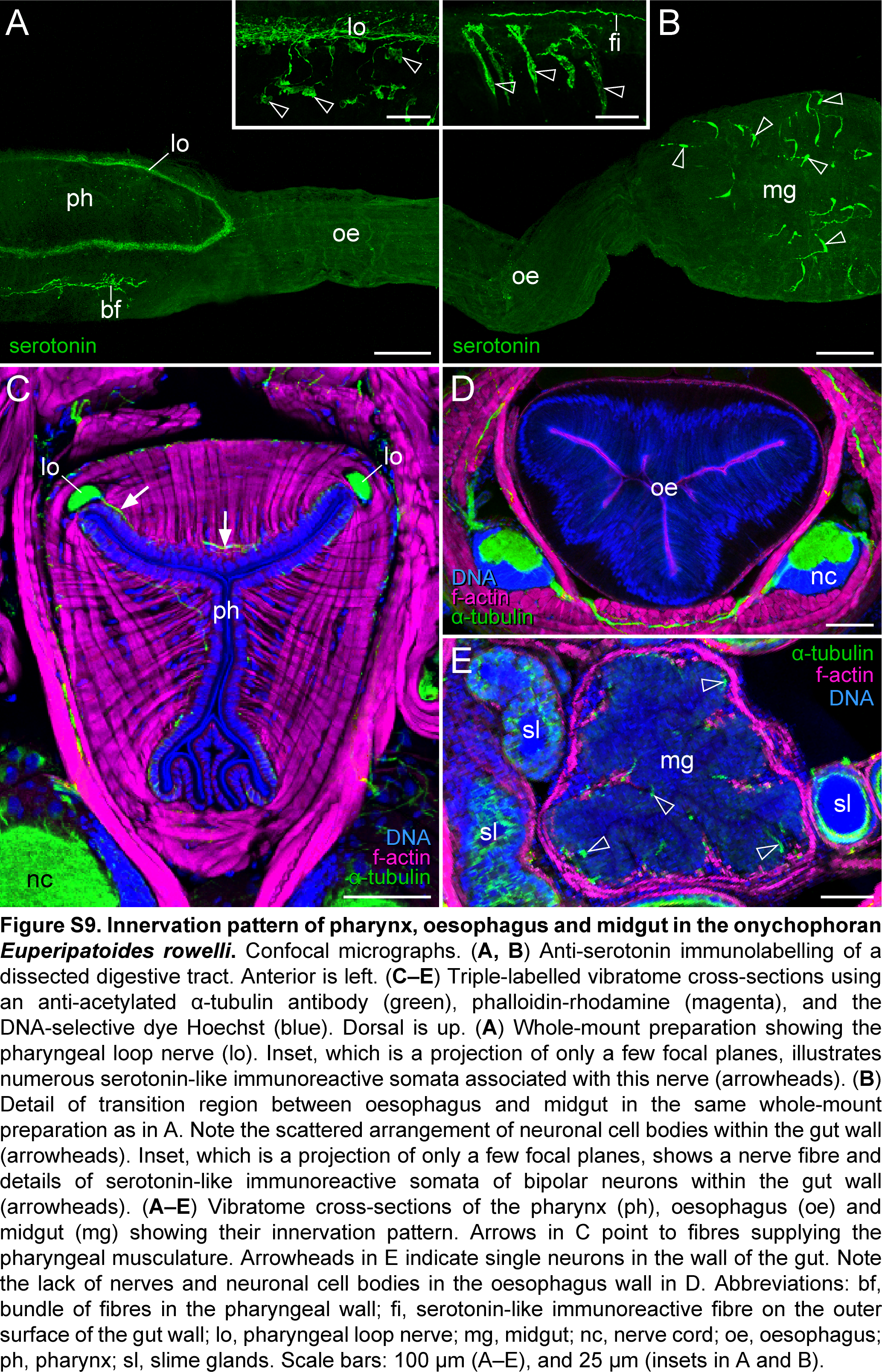

Supplement: Figure S9 — Innervation pattern of pharynx, oesophagus and midgut in the onychophoran Euperipatoides rowelli. Version for the colour-blind. Confocal micrographs. (A, B) Anti-serotonin immunolabelling of a dissected digestive tract. Anterior is left. (C–E) Triple-labelled vibratome cross-sections using an anti-acetylated α-tubulin antibody (green), phalloidin-rhodamine (magenta), and the DNA-selective dye Hoechst (blue). Dorsal is up. (A) Whole-mount preparation showing the pharyngeal loop nerve (lo). Inset, which is a projection of only a few focal planes, illustrates numerous serotonin-like immunoreactive somata associated with this nerve (arrowheads). (B) Detail of transition region between oesophagus and midgut in the same whole-mount preparation as in A. Note the scattered arrangement of neuronal cell bodies within the gut wall (arrowheads). Inset, which is a projection of only a few focal planes, shows a nerve fibre and details of serotonin-like immunoreactive somata of bipolar neurons within the gut wall (arrowheads). (A–E) Vibratome cross-sections of the pharynx (ph), oesophagus (oe) and midgut (mg) showing their innervation pattern. Arrows in C point to fibres supplying the pharyngeal musculature. Arrowheads in E indicate single neurons in the wall of the gut. Note the lack of nerves and neuronal cell bodies in the oesophagus wall in D. Abbreviations: bf, bundle of fibres in the pharyngeal wall; fi, serotonin-like immunoreactive fibre on the outer surface of the gut wall; lo, pharyngeal loop nerve; mg, midgut; nc, nerve cord; oe, oesophagus; ph, pharynx; sl, slime glands. Scale bars: 100 µm (A–E), and 25 µm (insets in A and B). (TIF) [file pone.0059090.s009.tif]
